# The Transcriptomic Landscape of *Cupriavidus metallidurans* CH34 Acutely Exposed to Copper

**DOI:** 10.3390/genes11091049

**Published:** 2020-09-04

**Authors:** Laurens Maertens, Natalie Leys, Jean-Yves Matroule, Rob Van Houdt

**Affiliations:** 1 Microbiology Unit, Interdisciplinary Biosciences, Belgian Nuclear Research Centre (SCK CEN), 2400 Mol, Belgium; laurens.maertens@sckcen.be (L.M.); nleys@sckcen.be (N.L.); 2 Research Unit in Microorganisms Biology (URBM), Narilis Institute, University of Namur, 5000 Namur, Belgium; jean-yves.matroule@unamur.be

**Keywords:** copper, differential RNA-sequencing, *Cupriavidus metallidurans*, differential gene expression, transcription start site, gene regulation, sRNA

## Abstract

Bacteria are increasingly used for biotechnological applications such as bioremediation, biorecovery, bioproduction, and biosensing. The development of strains suited for such applications requires a thorough understanding of their behavior, with a key role for their transcriptomic landscape. We present a thorough analysis of the transcriptome of *Cupriavidus metallidurans* CH34 cells acutely exposed to copper by tagRNA-sequencing. *C. metallidurans* CH34 is a model organism for metal resistance, and its potential as a biosensor and candidate for metal bioremediation has been demonstrated in multiple studies. Several metabolic pathways were impacted by Cu exposure, and a broad spectrum of metal resistance mechanisms, not limited to copper-specific clusters, was overexpressed. In addition, several gene clusters involved in the oxidative stress response and the cysteine-sulfur metabolism were induced. In total, 7500 transcription start sites (TSSs) were annotated and classified with respect to their location relative to coding sequences (CDSs). Predicted TSSs were used to re-annotate 182 CDSs. The TSSs of 2422 CDSs were detected, and consensus promotor logos were derived. Interestingly, many leaderless messenger RNAs (mRNAs) were found. In addition, many mRNAs were transcribed from multiple alternative TSSs. We observed pervasive intragenic TSSs both in sense and antisense to CDSs. Antisense transcripts were enriched near the 5′ end of mRNAs, indicating a functional role in post-transcriptional regulation. In total, 578 TSSs were detected in intergenic regions, of which 35 were identified as putative small regulatory RNAs. Finally, we provide a detailed analysis of the main copper resistance clusters in CH34, which include many intragenic and antisense transcripts. These results clearly highlight the ubiquity of noncoding transcripts in the CH34 transcriptome, many of which are putatively involved in the regulation of metal resistance.

## 1. Introduction

Environmental pollution with toxic metals due to anthropogenic activities is an internationally growing concern [1,2,3]. The exposure and the risk of elevated concentrations of these pollutants in the environment can lead to bioaccumulation and harmful effects [4,5,6,7], which are facilitated by the high toxicity and recalcitrance of some metals [8,9]. Consequently, monitoring tools for metal accumulation in natural environments such as soils and water bodies are needed. Physicochemical analysis techniques, while accurate and sensitive, often fail to chart the bioavailability and the toxicity of the polluting components [10,11,12]. At the same time, microorganisms show great promise as biosensors to quantify the bioavailable fraction of heavy metals such as copper [13], an essential trace element that is highly toxic when overly abundant [14]. In addition, microorganisms can be used to combat environmental contamination with heavy metals in a process called bioremediation [3,8,15,16]. Bioremediation has been the focus of extensive research in recent years as a clean and efficient alternative to conventional strategies (reviewed in Tabak et al., 2005 [17] and Akcil et al., 2015 [18]).

*Cupriavidus metallidurans* strains are exemplary β-proteobacteria in metal-contaminated industrial environments [19]. Type strain CH34 was first isolated from a decantation basin in a zinc factory near Engis, Belgium, in 1976 [14,20]. It was quickly shown to encode resistance mechanisms to a wide range of metals [21] and has become a model organism to study heavy metal resistance (HMR) in bacteria [19]. Copper resistance in strain CH34 is mediated by multiple cooperating Cu detoxification systems; *copF* and *copA*_1_*B*_1_*C*_1_*D*_1_ encode a Cu efflux P-type ATPase and a periplasmic detoxification system, respectively, and are part of the extensive 21-gene *cop* cluster. The neighboring *silDCBA* cluster encodes a heavy metal efflux- resistance nodulation division (HME-RND) driven system. These gene clusters are encoded on the pMOL30 megaplasmid, and homologous systems can be found on its chromosome and chromid (Table 1). In addition, many more accessory genes may play a role in Cu resistance. The integration of these systems brings about a minimum inhibitory concentration of 3 mM Cu^2+^, three times higher than that of *Escherichia coli* K38 [22,23]. A detailed description of Cu resistance in strain CH34 can be found in Mergeay and Van Houdt [19].

In a previous study, biosensors for metals such as Pb^2+^, Zn^2+^, and Cu^2+^ have been developed based on *Cupriavidus* strains [24,25]. These biosensors function via a transcriptional fusion of metal-specific promotor regions to a *luxCDABE* luciferase gene cluster, which allows for the emission of a measurable bioluminescent signal upon translation (BIOMET^®^ system). The strength of this signal is proportional to the biologically available fraction of a specific metal. *Cupriavidus*-based biosensors have been used for the characterization of bioavailable metal fractions in soil, sediments, mineral wastes, etc. [26,27,28]. In addition, the bioremediation potential of CH34, owing to its capacity for metal solubilization and biocrystallization, has been demonstrated in several experimental setups [24,29]. For instance, the deposition of ZnCO_3_ and CdCO_3_ crystals around the cell surface, both biologically catalyzed and via abiotic processes, can lead to efficient depletion of these toxic metal ions from the environment. *Cupriavidus* strains are especially interesting in the case of mixed pollution with metals and recalcitrant organic compounds, since they are often able to degrade a broad array of aromatic compounds [30,31]. However, the construction of bacterial strains able to efficiently cope with mixed pollutions remains challenging [32].

In order to understand, optimize, and control the molecular processes underlying the above mentioned applications, it is vital to understand their regulation. Indeed, an elaborate network of sigma factors [33,34] and transcriptional regulators [35,36] has been found to play an important role in CH34. However, new paradigms in bacterial gene regulation highlight the role of small regulatory RNAs (sRNAs), acting at a (post-)transcriptional level in disparate stress responses [37,38,39]. For instance, sRNAs are involved in a plethora of biological processes, including virulence [40,41,42,43,44,45,46,47], antibiotic resistance [48,49,50,51,52] and mobile genetic elements [53] as well as oxidative and metal stress [54,55,56,57,58,59,60] and the degradation of organic compounds [61].

Thus, in order to fully understand the intricate response of *C. metallidurans* CH34, a highly detailed map of its transcriptome is a basic necessity. Although its genome is fully sequenced and annotated [31], and transcriptome data in response to metal stress are available [22,34], these data do not allow analyzing pertinent features such as transcription start sites (TSSs), 5′ untranslated regions (5′ UTRs), RNA processing sites (PSSs) and regulatory RNAs. Therefore, we used a tagRNA-sequencing (tagRNA-seq) approach, a modified RNA-seq method that is based on the differential labeling of 5’ RNA ends and that enables whole transcriptome sequencing with the discrimination of primary from processed 5’ RNA ends [62,63]. The ability of tagRNA-seq to annotate TSSs has been well established in recent years, and it is commonly exploited in research on regulatory features of the bacterial transcriptome. Copper was used as imposed stress, since CH34′s copper resistance mechanism is unique in respect to its complexity compared to others [19], and the putative functions of sRNAs in the bacterial copper response remain insufficiently studied. We provide an overview of differentially expressed genes, a reannotation of the CH34 genome, and an analysis of the regulatory landscape.

## 2. Materials and Methods

### 2.1. Bacterial Strains, Media, and Culture Conditions

*C. metallidurans* CH34 [20] was routinely grown in Tris-buffered mineral medium (MM284) [21] supplemented with 2 g/L gluconate on an orbital shaker at 180 rpm in the dark at 30 °C. MM284 agar plates were prepared by adding 2% agar (Thermo Scientific, Oxoid, Hampshire, UK) to the liquid medium.

### 2.2. RNA Extraction

Bacterial cultures were prepared in triplicate by inoculating pre-warmed MM284 medium with several *C. metallidurans* CH34 colonies and growing the cells at 30 °C on an orbital shaker at 120 rpm. After 48 h, cultures were diluted with pre-warmed 284 MM medium to an OD_600_ of 0.1 and grown to an OD_600_ of 0.4. At this point, either CuSO_4_ was added to a final concentration of 400 µM (copper condition) or a corresponding volume of H_2_O (control) was added. After 10 min of exposure, cultures were put on ice and washed twice with a chilled 10 mM MgSO_4_ solution at 4 °C. After washing, bacterial pellets were flash frozen and stored at −80 °C until RNA extraction. RNA extraction was performed with the *mir*Vana™ miRNA Isolation kit (Invitrogen™, Carlsbad, CA, USA). Lysis was performed by resuspending the bacterial pellet in 50 µL of a 3 mg/mL lysozyme solution (Sigma Aldrich, Saint Louis, MO, USA) and incubating it at room temperature for 15 min. After lysis, a volume of 500 µL Lysis/Binding buffer was added. The protocol for total RNA extraction was followed without enrichment for small RNAs. Total RNA quality was measured by Agilent 2100 Bioanalyzer using Nano chips. Only samples with a RIN value above 9 were accepted for sequencing.

### 2.3. TagRNA-Sequencing Protocol

A tagRNA-seq protocol [64,65,66] was performed by Vertis Biotechnologie AG (Freising, Germany) (Supplementary Figure S1). The complementary DNA (cDNA) libraries were constructed in the following manner: the 5′ Illumina TruSeq sequencing adapter with a CTGAAGCT tag was ligated to the 5′ monophosphate groups of rRNA-depleted RNA samples. Subsequently, samples were treated with Terminator Exonuclease (TEX, Lucigen, Middleton, WI, USA) to remove RNAs with non-ligated 5′ monophosphate ends. Then, RNA 5′-polyphosphatase (5′PP, Lucigen) was used to convert 5′ triphosphate groups into 5′ monophosphate groups, and the 5′ Illumina TruSeq sequencing adapter with TAATGCGC was ligated to the newly formed 5′ monophosphate groups. RNA samples were then fragmented, and a 3′ sequencing adapter was ligated to the RNA fragments’ 3′ ends. First-strand cDNA synthesis was performed using M-MLV reverse transcriptase and the 3′ adapter as primer. The resulting cDNA was purified, ligated with a 5′ sequencing adapter, and PCR amplified using a proofreading enzyme. The cDNA was then purified using the Agencourt AMPure XP kit (Beckman Coulter Genomics, Chaska, MN, USA) and analyzed by capillary electrophoresis. The size fraction of 160–500 bp was eluted and sequenced on an Illumina NextSeq 500 system using 75 bp read length. The different sequencing adapters in combination with RNA fragmentation resulted in three read libraries for each sample: reads originating from the 5′ end of 5′-monophosphate (5′-P) RNA molecules (processed start sites, PSS reads), reads originating from the 5′ end of 5′-triphosphate (5′-PPP) RNA molecules (transcription start sites, TSS reads), and reads originating from non-tagged RNA fragments enabling coverage over the entire transcript. For each sample, ca. 20 million reads were generated. Adapter sequences were trimmed from all read libraries using Trimmomatic version 0.36 [67], and raw read quality was assessed using FastQC version 0.10.0 (https://www.bioinformatics.babraham.ac.uk/projects/fastqc/). Reads were aligned to the *C. metallidurans* CH34 reference genome (National Center for Biotechnology Information (NCBI) accession numbers NC_007971.1, NC_007972.1, NC_007973.1, and NC_007974.1) with bowtie2 [68] using default parameters. Within each biological replicate and condition, coverage values were similar over all four replicons. The RNA-seq datasets generated and analyzed for this study are available from the NCBI Sequence Read Archive (SRA) under accession number PRJNA639913.

### 2.4. Differential Gene Expression Calculation and Functional Enrichment Analysis

Differential gene expression was calculated by merging PSS, TSS, and unassigned libraries of each sample using samtools merge [69]. Coverage of annotated genes of the resulting bam files was performed with the featureCounts function of the Rsubread package [70] for R version 3.6.1. Options ---isStrandSpecific and –fraction were set to TRUE, and option --countMultiMappingReads was set to FALSE. Differential gene expression of the resulting count matrix was accomplished using edgeR [71,72] and limma [73], using a Benjamini–Hochberg [74] approach to control for Type 1 statistical errors. Functional enrichment analysis was performed using the eggNOG [75] classification of annotated genes, as found on the MaGe platform [76], and the MLP package for R, version 1.34.0, was used for *p*-value calculation.

### 2.5. Transcriptional Start Site Profiling

TSSs were detected using an in-house Python script (Python version 2.7, TSS_res_finder.py in Supplementary Data). The 5′ most base of all reads in the TSS read library was selected using bedtools (version 2.19.1) [77], and subsequent analyses were performed on the single base position to which the read was reduced. Reads of triplicate TSS libraries from the same condition were combined into a single library for further analysis. The PSS and the unassigned libraries were not used for TSS profiling. Coverage values of a sliding window with an arbitrary width of 5 nt were calculated for all nucleotide positions. If the coverage value of a sliding window exceeded 50 reads relative to a read library size of two million reads, the coverage-weighted average TSS position within this sliding window was calculated.

### 2.6. 5′ and 3′ RACE Protocol

The 5′ and the 3′ rapid amplification of cDNA ends (5′/3′ RACE) was performed using the SMARTer^®^ 5′/3′ RACE kit (Takara Bio, Saint-Germain-en-Laye, France) using the standard protocol. Total RNA was obtained as described in the section “RNA extraction”. A list of primers can be found in Supplementary Table S1 (first 14 primers). Random primers were used for 5′-first-strand cDNA synthesis. In a second round of RACE experiments, RNA was poly(A)-tailed for 20 min using poly(A)-polymerase (Invitrogen™) before proceeding with 5′ and 3′ RACE protocol. Primer sequences can be found in Supplementary Table S1 (last 20 primers).

### 2.7. Statistics

The functional enrichment of eggNOG classes with disparate TSS classes was calculated using Fischer’s exact test with subsequent Benjamini–Hochberg correction for multiple testing [74]. R version 3.6.1 was used for all statistical analyses. Adjusted *p*-values below 0.05 were considered statistically significant.

## 3. Results and Discussion

### 3.1. Differential Gene Expression Analysis

First, the transcriptomic response to acute non-lethal Cu^2+^ exposure was analyzed. This yielded 352 coding sequences (CDSs) that were significantly up- (155) or downregulated (197) in the presence of Cu^2+^, amounting to 5.15% of the total number of CDSs. Thirty-two and nine of these were located on pMOL30 and pMOL28, respectively (Supplementary Figure S2). CDSs with false discovery rate (FDR) values lower than 0.05 and logFC values higher than 1 or lower than −1 were considered differentially expressed. In comparison with the previous microarray analysis (403 up and 373 down), the overlap was limited with 40 up- and 25 downregulated CDSs shared between both analyses. However, it is important to acknowledge the difference in dose (100 vs. 400 µM) and exposure time (30 min vs. 10 min), as both factors may have strongly influenced the transcriptomic response measured [22].

**Figure 1:**
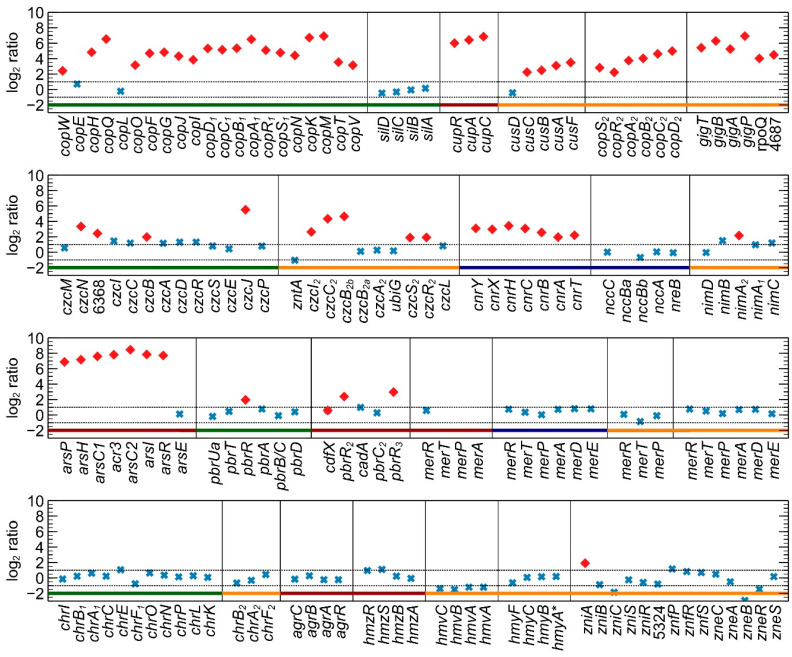
Differential gene expression of metal resistance genes from *C. metallidurans* CH34 exposed for 10 min to 400 µM Cu^2+^. Significant (red diamond; *p* < 0.05) and non-significant (blue cross) log_2_ ratios are shown for known metal resistance genes located on the chromosome (red line under graph), chromid (orange), pMOL28 (blue), and pMOL30 (green). Dotted lines correspond to −1 and 1, respectively.

The functional relevance of differentially expressed CDSs was explored using functional categories from the eggNOG classification system and the MLP package. Classes P (inorganic transport and metabolism), E (amino acid transport and metabolism), and O (posttranslational modification, protein turnover, and chaperones) were significantly enriched (Supplementary Table S2). In the previous microarray analysis [22], eggNOG classes P and M (cell wall/membrane/envelope biogenesis) were significantly overrepresented. This observation again underlines the adjustability of the copper-exposed transcriptome as a response to differences in dose and response time. The strong enrichment of class P was mostly because of the upregulation of many HMR systems (Figure 1). As mentioned previously, the three main Cu detoxification systems are encoded by the *cop*_1_ and the *sil* clusters on the pMOL30 plasmid. With respect to the Cu resistance gene clusters (Table 1), the whole *cop*_1_ cluster was upregulated, except *copE* (unknown function) and *copL* (cys-rich cytosolic protein) (Figure 1). The neighboring *ubiE* (Rmet_6137) and *ubiE*_2_ (Rmet_6131) genes, encoding methyltransferases, were also upregulated. Interestingly, no differential expression was observed for the *silDCBA* cluster (Rmet_6133–6136). The latter is mainly induced by Ag^+^, but *silA*, *silC* and *silD* have been reported to be induced by Cu^2+^ after 30 min of exposure [19]. As indicated above, each of the main Cu detoxification systems has a homologous system on the chromosome or the chromid (Table 1). All of them, P-type ATPase (*cupRAC*; Rmet_3523–3525), periplasmic detoxification system (*copD*_2_*C*_2_*B*_2_*A*_2_*R*_2_*S*_2_; Rmet_5668–5672), and HME-RND-driven system (*cusDCBAF*; Rmet_5030–5034), were completely upregulated, with the exception of *cusD*. This observation was surprising, since it has been hypothesized that the *cop*_2_ cluster on the chromid is involved in a late response to relatively low Cu^2+^ concentrations [78]. Our results clearly show a prompt response of these clusters to acute Cu stress.

Cross-regulatory interactions, i.e., the induction of other HMR mechanisms by Cu, were observed for at least six clusters. The 8-gene *ars* cluster on the chromosome (Rmet_0327–0334), encoding resistance mechanisms against As, was completely upregulated (except *arsE*). It has previously been shown that the *ars* cluster is upregulated after exposure to As^3+^, Pb^2+^, Zn^2+^, Co^2+^, Cd^2+^, and Se^6+^ [22], but a link with Cu exposure has thus far not been found. However, it has been shown that the regulator ArsR can bind Cu^2+^ [79]. Cross-regulation with resistance mechanisms against Cd^2+^, Zn^2+^, and Co^2+^ toxicity was also observed via the upregulation of *czcB*_1_, *czcJ*_1_, and *czcN*_1_, with *czcJ*_1_ also shown to be upregulated after 30 min Cu exposure [22]. The chromid-borne *czc*_2_ cluster (Rmet_4464–4469, Rmet_4595–4597) was partly upregulated (Figure 1 and Supplementary Figure S2). The response regulators CopR_1_ and CopR_2_ are highly similar to CzcR_1_ and CzcR_2_ (roughly 60% amino acid identity), which could elicit cross-regulation. Furthermore, the regulon of response regulators could have been broader than their co-localized targets [80,81,82]. Enhanced transcription of the CzcRS two-component system after Cu exposure was also noted in *Pseudomonas aeruginosa* [83]. Induction of several genes involved in Pb^2+^ resistance was also detected, with three copies of the *pbrR* gene encoding a MerR-type regulator being upregulated. Two copies are located on the chromosome (Rmet_2302, Rmet_3456), but lead resistance is mainly attributed to the *pbrUTRABCD* cluster on pMOL30 (Rmet_5944–5949). However, none of the other genes in this cluster were overexpressed. Additional cross-regulation was observed in the complete upregulation of the *cnr* cluster on pMOL28 (Rmet_6205–6211), which encodes resistance against Ni^2+^ and Co^2+^. It has previously been observed that the transcriptomic responses of CH34 to Cu^2+^, Co^2+^, and Ni^2+^ overlap considerably [22]. Finally, the *gig* cluster on the chromid (Rmet_4682–4687) was completely and strongly induced. This cluster was found to be upregulated by Au^3+^ [84] and Ag^+^ [22] in a previous study and is controlled by the extracytoplasmic function (ECF) RpoQ sigma factor [84], which is also overexpressed after acute Cu stress. RpoQ has also been implicated in the resistance to Cd^2+^ and in the regulation of thiol/sulfide metabolism in CH34 [34].

A second eggNOG class enriched upon Cu exposure was class O, comprising posttranslational modification, protein turnover, and chaperones, which can be associated with the production of reactive oxygen species and antioxidant depletion elicited by Cu toxicity [85]. In agreement with previous studies [86], even after 10 min of Cu exposure, considerable changes were observed in the expression of genes involved in redox cycling and their regulatory mechanisms. Redox sensors *oxyR* [87] and *soxR* [88] were both upregulated. Curiously, CH34 lacks the homologs of their regulatory counterparts *oxyS* and *soxS*, thus the exact mechanisms of transcriptional regulation are unknown and may differ from canonical mechanisms in, e.g., *Escherichia coli* [31]. In addition to *oxyR* and *soxR*, several peroxidase genes, such as *katA*, *aphC*, and *aphD* but not *sodB* and *sodC*, genes involved in thioredoxin and glutaredoxin metabolism and Fe-S cluster assembly as well as *ohrR* (coding for a transcriptional regulator of organic hydroperoxide resistance [89,90]) and *dpsA* (encoding a DNA-binding protein associated with oxidative stress protection), were upregulated (Supplementary Table S3, Sheet “DGE”). Antioxidants such as glutathione as well as Fe-S cluster-containing proteins with roles as transcriptional regulators, catalytic enzymes, or oxidative stress sensors have been shown to play an important role in relieving oxidative stress [91,92,93,94,95,96,97]. Interestingly, this response to oxidative stress was not observed in a previous experiment with a longer exposure time (30 min) at a lower concentration (100 µM) [22], suggesting that oxidative stress is only elicited at higher Cu concentration or is rapidly counteracted. Several genes related to protein turnover and protein chaperoning were differentially expressed, which, together with the overrepresentation of class E (amino acid transport and metabolism), indicates the need to reconfigure the proteome in order to protect the cell from Cu toxicity. One of two copies of the chaperone *clpB* [98] was upregulated, as were *dnaK* [99], the *hslUV* operon (encoding a bipartite ATP-dependent protease [100]), *mucD* (periplasmic endopeptidase) [101], *prlC* (oligopeptidase) [102], and *ftshH* (metalloprotease) [103,104] (Supplementary Table S3, sheet “DGE”). The importance of protein turnover, both as a direct result of and as an active response to Cu stress, has been well established [105,106]. In addition, several upregulated pathways are associated with amino acid metabolism (Supplementary Table S3, sheet “DGE”). For instance, the two *cys* clusters involved in cysteine metabolism were almost completely upregulated [107]. The importance of the cysteine metabolism, which is intimately linked to the use of cellular S pools, has been implied in Cu resistance in other studies [86,108,109,110,111]. A prime example is the need for L-cysteine in the biosynthesis of glutathione, of which the anabolic pathways were upregulated in the copper condition. Next to changes in S-metabolism, the transcriptome governing the metabolism of several other amino acids was also altered by exposure to Cu (Supplementary Table S3, sheet “DGE”).

### 3.2. Transcriptional Start Site Profiling

#### 3.2.1. General Characteristics of Detected TSSs

In a next step, the tagRNA-seq data were used to identify and probe the type of the 5′ ends of RNAs in order to obtain a global snapshot of the transcriptional organization in *C. metallidurans* CH34 and to scrutinize the impact of Cu stress on the RNA landscape. The transcription start site (TSS) detection algorithm, as described in Materials and Methods, was fine-tuned to annotate the 7500 most highly expressed TSSs in both the control and the copper condition. This number was roughly based on the number of annotated genes and pseudogenes in CH34 (6514). These TSSs were divided into primary, secondary, internal, antisense, and orphan TSS according to their location, similar to previous publications [112] with minor modifications (Supplementary Figure S1). A primary TSS (pTSS) is the main TSS of a gene or operon and is located within 200 bp upstream of a start codon. It is expressed at least twice as strongly as the second most highly expressed TSS within those 200 bps. The remaining TSSs in this region were classified as secondary TSSs (sTSSs). An internal TSS (iTSS) is located within and on the coding strand, while an antisense TSSs (aTSS) is located on the non-coding strand of a CDS or within 100 bp upstream of its start codon. The orphan TSSs (oTSSs) are not associated with CDSs. An overview of this classification for every replicon is shown in Table 2. As TSS classification was prioritized via the following cascade: pTSS > sTSS > iTSS > aTSS > oTSS; there is no overlap between the different classes. In addition, the tagRNA-seq data was verified with 5′ and 3′ RACE of selected transcripts, including *copA*_1_ messenger RNA (mRNA) and several antisense and orphan transcripts (see below) (Supplementary Figure S3). Similarly, the previously identified TSSs of *cnrC*, *czcI*, and *pbrA* were also detected and confirmed by the tagRNA-seq results [113,114,115], both of which corroborate the validity of the TSS identification with the tagRNA-seq procedure.

#### 3.2.2. Primary TSSs

**Figure 2:**
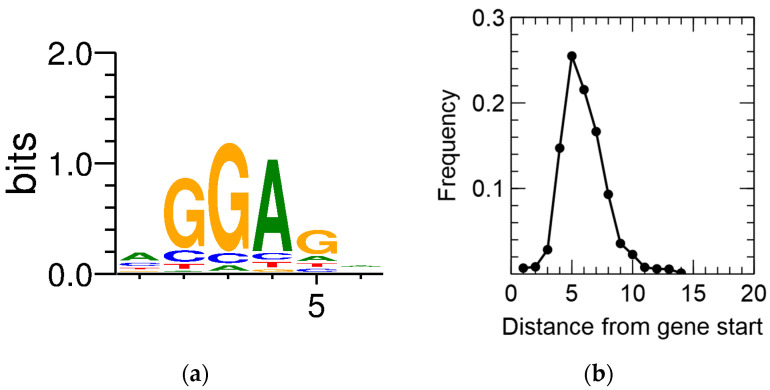
Ribosome-binding site sequence motif (**a**) and spacer length distribution (**b**) of *C. metallidurans* CH34.

Primary TSSs were detected for 2422 CDSs, amounting to 35.8% of all annotated CDSs. In a first step, this information was combined with the output of the recently developed GeneMarkS-2 gene prediction tool [116]. CDSs for which no pTSS was detected and that contained an iTSS between the original start codon and the newly predicted one, being preceded by a predicted ribosome-binding site (RBS), were scrutinized. This resulted in the putative reannotation of 182 CDSs. In addition, the consensus RBS and the spacer length were derived (Figure 2).

Four cases were related to metal resistance. First, related to copper, *copM* encoding a 136 aa uncharacterized pre-protein was putatively re-annotated to be 114 aa. The cleavage site was similar for both, i.e., the processed proteins were identical, but the signal peptide prediction was much better for the newly annotated CDS (0.82 vs. 0.66; SignalP-5.0) (Supplementary Figures S4 and S5). In addition, *cupC* encoding 133 aa Cu chaperone was putatively reannotated to be 66 aa (Supplementary Figures S4 and S6). Related to the *czc* cluster involved in cadmium, zinc, and cobalt resistance, *czcN* (273 aa; 30.1 kDa predicted molecular weight) encoding a membrane-bound isoprenylcysteine carboxyl methyltransferase was putatively reannotated to be 216 aa (23.7 kDa predicted molecular weight) (Supplementary Figures S4 and S7). CzcN is homologous to NccN of *Alcaligenes xylosoxidans* 31 (reclassified as *C. metallidurans*), which was found to be a 23.5 kDA protein [117]. Finally, the *pbrD* gene coding for a Pb^2+^-binding protein [114] could be putatively reannotated based on both TSS identification and GeneMarkS-2 prediction (CDS and RBS) (Supplementary Figure S8). However, the new CDS is not in frame and codes for a 150 aa protein unrelated to PbrD. These results merit further study of its function and sequence, since *pbrD* appears not to be necessary for Pb^2+^ resistance and is absent in all other known lead-resistant bacteria [118]. Moreover, although it is heterologously expressed in *E. coli*, it does show Pb^2+^ absorption [119].

**Figure 3:**
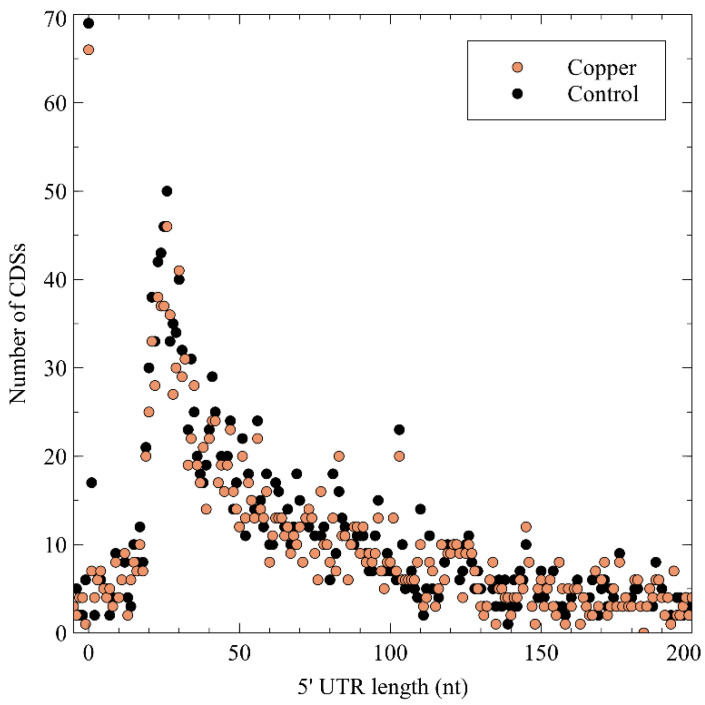
The 5′ untranslated region (UTR) length distribution in the *C. metallidurans* transcriptomes. CDS: coding sequence.

Next, the 5′ untranslated region (5′ UTR) lengths and their distribution were calculated (Figure 3), which showed a peak at a length of 26 nt, after which it tapered off. No difference was noted between the control and the Cu condition, indicating that Cu exposure does not entail a global measurable bias in the 5′ UTR length of expressed genes. A curious observation was the high number of leaderless mRNA (4.67% of all pTSSs), defined as those mRNAs with a 5′ UTR length shorter than 5 nt. Because of the lack of a 5′ UTR, these transcripts are translated via non-canonical mechanisms. Similar observations have been made in *Burkholderia cenocepacia* [120], *Streptomyces coelicor* [121], *Salmonella enterica* [122], *Caulobacter crescentus* [123,124], and *Helicobacter pylori* [125,126]. A functional enrichment analysis showed that leaderless mRNAs was significantly enriched in eggNOG class K (transcription), which has already been observed in many bacterial species [127], and class L (replication, recombination, and repair). Several leaderless mRNAs coding for transcriptional regulators were also differentially expressed in the presence of Cu^2+^, including *arsR*, *ompR*, *ohrR*, and *zniR*. In addition, *copJ*, *merP*, *rpoN*, and *czcI*_2_ were transcribed into leaderless mRNAs. The prevalence of leaderless mRNAs has been shown to be higher in organisms with an earlier evolutionary age, and has been linked to extreme habitats [127,128].

Finally, to complete the promoter information, the consensus sequence in a region of 100 nt around each pTSS was identified with Improbizer [129], which allowed us to weight the location of detected motifs. On the chromosome, the chromid, and the megaplasmid pMOL30, a TAnAAT consensus motif was detected around the −10 position, generally flanked by GC-rich stretches (Supplementary Figure S9). At the −35 position, a TTGACA-like motif with higher variability was detected, again flanked by short GC-rich stretches (Supplementary Figure S9). It is possible that CDSs preceded by these consensus motifs, which are similar in location and sequence to those found in *E. coli*, are under transcriptional control of the two housekeeping σ^70^-factors *rpoD*_1_ (Rmet_2606) and *rpoD*_2_ (Rmet_4661) [31]. Interestingly, although pMOL28 and pMOL30 do not contain essential housekeeping genes, TAnAAT consensus motifs were found on pMOL30 but not pMOL28, indicating a difference in sigma factor recruitment for the initiation of pMOL28 and pMOL30 gene transcription. Noteworthy, in contrast with pMOL30, which does not encode any sigma factors, pMOL28 encodes the ECF sigma factor CnrH. However, it is only involved in transcription of the *cnr* locus [113]. In addition, it has been shown that none of the remaining 10 ECF sigma factors, which are encoded on the chromosome (6) and the chromid (4), are necessary for the upregulation of any of the operons responding to metal shock [34]. Nevertheless, the involvement of non-plasmidic sigma factors in the transcription of plasmidic genes is not unexpected. On pMOL30, most CDSs preceded by a TAnAAT motif have an unknown function, but some genes involved in metal resistance are preceded by a TAnAAT-like pattern (*czcE*, *pbrT*, *ubiE*, and *copM*). Further determination of the functions of the remaining hypothetical proteins on both pMOL28 and pMOL30 may provide conclusive insights in this matter.

#### 3.2.3. Secondary TSSs

Alternative mRNAs deriving from secondary TSSs were detected for 623 CDSs. Among the genes differentially expressed under Cu stress, a high number of regulatory proteins was readily apparent, including the main regulators of pMOL30-based (*copR*_1_) and chromid-based periplasmic (*copR*_2_) Cu detoxification systems as well as *ohrR* (Rmet_3619), *zniS* (Rmet_5322), *bzdR* (Rmet_1223), *iscR*, *rpoH*, and *czcR*_2_. In addition, several genes involved in HMR and stress responses were transcribed from alternative TSSs (Supplementary Table S3, Sheet “sTSS”). Alternative TSSs have been reported in several bacterial and archaeal species [130,131,132] and have been linked to differences in translational efficiency and mRNA stability [133]. Longer 5′ UTRs enable more extensive post-transcriptional regulation, e.g., via sRNAs, and can contain riboswitches that respond to various environmental cues [134]. Interestingly, many mRNAs coding for sigma factors showed multiple TSSs, including *rpoA*, *rpoD*_1_, *rpoE*, *rpoH*, *rpoI*, *rpoK*, and *rpoM* (Supplementary Figure S10), which could have an impact on the transcription of genes associated with a particular sigma factor.

#### 3.2.4. Intragenic TSSs

The second most abundant TSS class after the pTSSs was that of the iTSSs. As all TSSs were derived from primary 5′-PPP-RNA, these iTSSs are unlikely to be the result of RNA degradation, e.g., by 5′-exonucleases, although some could be pTSSs or sTSSs due to misannotated CDSs, or transcriptional noise from spurious promotors. More importantly, iTSSs can also mark the start site of noncoding RNAs.

Even though the roles of the detected iTSSs are unclear, they are pervasive in the CH34 transcriptome. Functional enrichment analysis showed that, in the copper condition, eggNOG classes C, F, J, M, O, P, and T were enriched in iTSSs. In total, 55 of the 167 genes involved in metal resistance contained at least 1 iTSS, e.g., almost all *cop*_1_ genes (Supplementary Table S3, sheet “iTSS”) and follow-up functional analyses could provide more insights into their specific role in metal resistance. The iTSSs related to the Cu resistance mechanisms (*cop*_1_ and *sil* clusters on pMOL30) are provided and discussed in detail below (Section 3.2.7).

**Figure 4:**
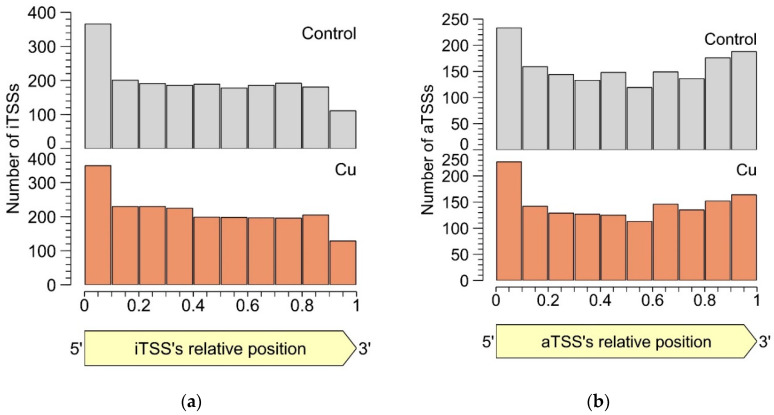
Percentile-wise distribution of iTSSs (**a**) and aTSSs (**b**) positions relative to the cognate CDS.

In both the control and the Cu condition, iTSSs were enriched in the first 10% of the encompassing CDS, while iTSSs were relatively depleted in the last 10% (Figure 4). This observation lends credibility to the hypothesis that many detected iTSSs are pTSSs or sTSSs to misannotated CDSs. However, these were not included in our re-annotation, as the current approach does not allow discriminating between the original and the putative new start codon. Of course, these uncertainties could be addressed with additional protein-based experiments such as proteome profiling (although not straightforward as the absence of a longer protein over the presence of a shorter one needs to be shown), but this is out of the scope of this study. Since there is currently no evidence that iTSSs in the first percentile are linked more to coding RNAs than in other percentiles, we conclude that many iTSSs either mark the start site of noncoding RNA or are the result of transcriptional noise.

#### 3.2.5. Antisense TSSs

Similar to intergenic TSSs, antisense TSSs are ubiquitously found in the CH34 transcriptome. In total, 2129 aTSSs were associated with 692 CDSs, indicating that many mRNAs can putatively be complexed by multiple antisense transcripts. Of all aTSSs, 408 showed a logFC smaller than −1 or greater than 1, indicating differential expression. Six differentially expressed aTSSs were validated by 5′ RACE experiments (Supplementary Table S1 and Supplementary Figure S3). It is generally assumed that aTSSs mark the start of noncoding transcripts, however, whether a detected antisense transcript has a (regulatory) function or is the result of transcriptional noise [135] needs to be determined case-by-case. Since antisense transcripts can obscure regulatory features of the sense transcript by perfect base pairing [136], the location of aTSSs relative to the 5′ and 3′ ends of the sense transcript was scrutinized (Figure 4b). There is a clear enrichment of aTSSs near the 5′ end of the sense CDS, with a less prominent enrichment near the 3′ end, indicating a putative regulatory role of the antisense transcripts.

**Figure 5:**
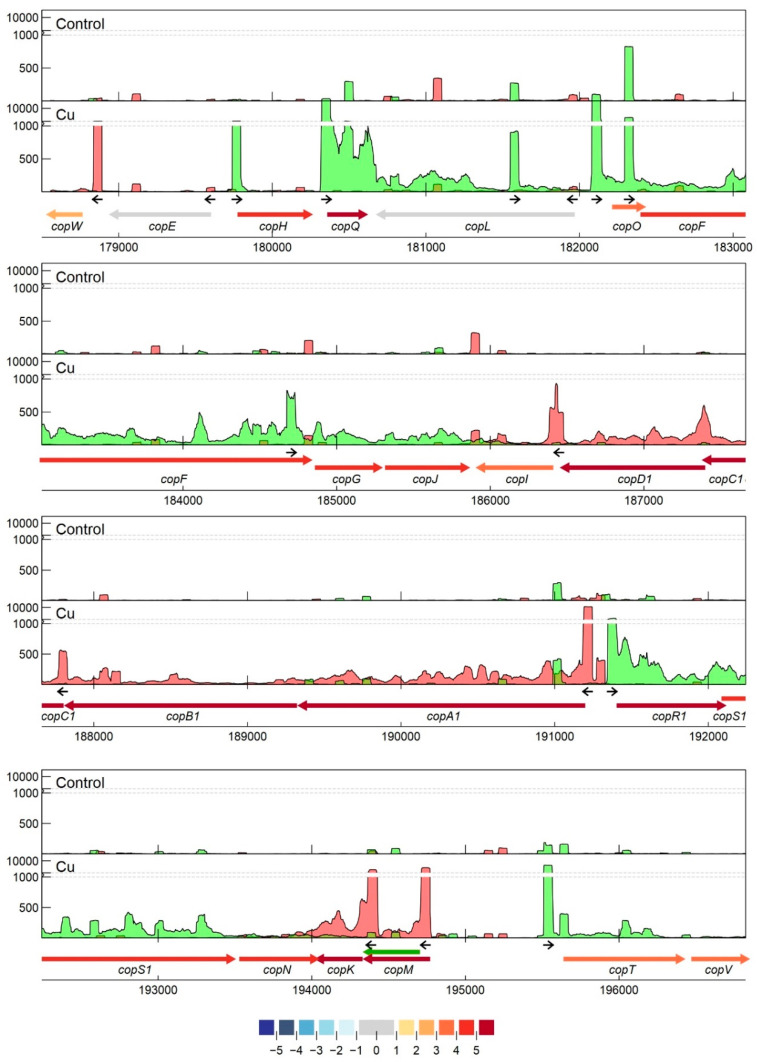
Transcription profile analysis of the *cop* cluster from *C. metallidurans* CH34 when exposed to copper. Combined TSS read counts of the three biological replicates for control (upper) and Cu condition (lower) are shown for positive (green) and negative (red) strands, with the *y*-axis containing a break pair (1000–1200) represented as striped grey lines. CDSs related to the *cop* cluster (coordinates for the pMOL30 region shown at the bottom) are colored based on their log_2_ fold change. The small black arrows indicate clearly identified primary and internal TSSs. The green arrow represents a re-annotated CDS.

**Figure 6:**
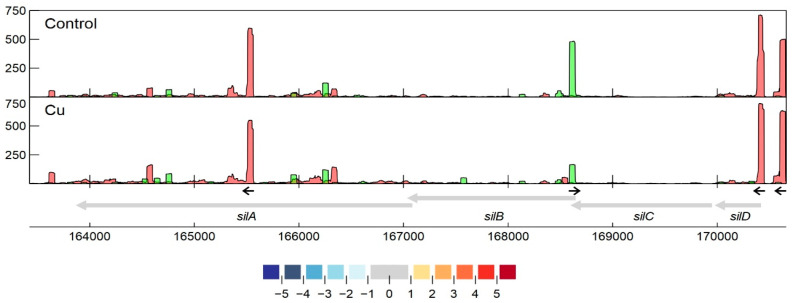
Transcription profile analysis of the *sil* cluster from *C. metallidurans* CH34 when exposed to copper. Combined TSS read counts of the three biological replicates for control (upper) and Cu condition (lower) are shown for positive (green) and negative (red) strands. CDSs related to the *sil* cluster (coordinates for the pMOL30 region shown at the bottom) are colored based on their log_2_ fold change. The small black arrows indicate clearly identified primary and internal TSSs.

Specifically related to copper resistance, aTSSs were found in *silB*, *copA*_1_, *copF*, *copG*, *copH*, *copL*, and *copM* (Figure 5 and Figure 6). The aTSSs in *silB* (nt position 168,587 on pMOL30) and *copL* (nt position 181,548 on pMOL30) were further selected for 3′ RACE validation (detailed in Section 3.2.7), indicating transcript lengths of approximately 700 and 500 bp, respectively (Supplementary Figure S3). In addition, an aTSS was found in *czcA*_1_ (Supplementary Figure S7).

Antisense TSSs were enriched in CDSs belonging to eggNOG classes L (replication, recombination, and repair), M (cell wall/membrane/envelope biogenesis), and T (signal transduction mechanisms) in the control condition, and classes J (translation, ribosomal structure and biogenesis), L, and M in the copper condition. Class T was only significantly overrepresented in the control condition, which could indicate that repression of translation via antisense transcription is common for CDSs related to signal transduction and partially relieved under copper stress. Many sigma factors, such as *rpoB* (Rmet_3334), *rpoD*_1_ (Rmet_2606), *rpoN*, *rpoB*, and *rpoS* (Rmet_2115), showed antisense transcription to a high extent (Supplementary Figure S10). The role of alternative sigma factors in the initiation of antisense transcription has been described before [137,138,139]; inversely, an interesting case of the regulation of the sigma factor RpoS itself by antisense and anti-antisense RNAs has been shown in *E. coli* [140].

#### 3.2.6. Orphan TSSs

In total, 578 TSSs were detected in intergenic regions unassociated with CDSs. These orphan TSSs could mark the start of transcripts resulting from the 5′ of mRNAs with exceptionally long 5′ UTRs, unidentified CDSs, often coding for short peptides [141], and trans-acting sRNAs [142], or they could result from transcription by spurious promoters.

First, the prevalence of long 5′ UTRs was investigated. A 200–300 nt region upstream of known start codons was scrutinized for oTSSs, and uninterrupted coverage from the oTSS to the start codon was manually verified. In this way, 126 oTSSs marked the 5′ of putative long 5′ UTRs. Interestingly, *hfq*, coding for a small RNA-binding protein that stabilizes sRNAs and modulates RNA–RNA interactions [143] showed a long 5′ UTR (209 bp) next to two other mRNA isoforms with 5′ UTRs of 25 and 137 bp. Relative expressions of these three isoforms (25, 137, and 209 nt 5′ UTR) were 6.1%, 81.1%, and 12.8% and 15.0%, 74.7%, and 10.3% for control and copper conditions, respectively. These disparate 5′ UTRs might have given rise to differences in post-transcriptional regulation of the *hfq* mRNA. While the relative coverage of each alternative TSS was quite similar in control and copper conditions, it is possible that stronger selection of differential TSSs exists in other (stress) conditions.

In a subsequent analysis, the existence of putative open reading frames (ORFs) downstream of oTSSs was studied. In total, 417 oTSSs were located upstream an ORF with ATG, GTG, or TTG as start codons and either with a minimum length of 150 nt or with homology to proteins in the non-redundant protein database. A list of these proteins with their inferred function, among which are several transporters, can be found in Supplementary Table S3, sheet “oTSS ORF BLAST”.

Consequently, the remaining 35 oTSS were neither located within 300 nt upstream of known CDSs nor associated with putative ORFs. These oTSSs were assumed to mark the 5′ end of noncoding RNAs. Eleven oTSSs showed a log_2_ fold change above 1 or below −1. As the expression of these oTSSs is affected by Cu stress, they are especially interesting as candidates for sRNAs with regulatory functions in the response to metal exposure. Three of them, based on interesting locations relative to known HMR genes, were experimentally validated by 5′ RACE (Supplementary Table S1 and Supplementary Figure S3). Two of these differentially expressed orphan transcripts (Supplementary Figure S11) were also experimentally validated by 3′ RACE (one at CHR1 nt position 3,784,309 and one at CHR2 nt position 147,562), indicating lengths of roughly 550 bp and 450 bp (Supplementary Figure S3). It must be mentioned that 3′ ends of coding and non-coding RNAs cannot be accurately inferred from tagRNA-seq coverage, hence the need for additional experimental validation (via 3′ RACE or other techniques).

#### 3.2.7. Detailed Analysis of *cop* and *sil* Clusters on pMOL30

**Figure 7:**
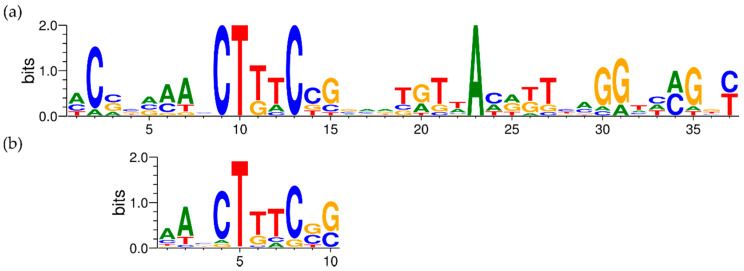
Conserved motifs 5′ of the *cop* cluster pTSSs according to MEME (**a**) and Improbizer (**b**).

As mentioned earlier, the *copVTKMNS*_1_*R*_1_*A*_1_*B*_1_*C*_1_*D*_1_*IJGFOLQHEW* cluster on pMOL30 harbors two of the three main Cu resistance systems. It is aided by the neighboring clusters *silDCBA*, *gtrM*_2_*A*_2_*B*_2_, *ompP*_2_, *ubiE*, and *ubiE*_2_. Homologous but less extensive combinations of Cu/Ag resistance mechanisms are widespread and are often associated with mobile genetic elements [144]. Although the *cop* cluster was almost completely induced by Cu^2+^, not all genes were induced to the same extent (Figure 1 and Figure 2). It has previously been shown that the transcriptomic response of some *cop* genes to Cu^2+^ stress is time-dependent [78], which indicates an additional level of regulatory complexity. Based on expression levels and TSS data, pTSSs were identified for *copW*, *copE*, *copH*, *copQ*, *copL*, *copO*, *copF*, *copGJ*, *copI*, *copC*, *copA*, *copR*_1_*S*_1_*N*, *copK*, *copM*, and *copTV*. The *copA* pTSS was validated with 5′ RACE. We investigated whether regulatory motifs could be found upstream of these pTSSs. Results using MEME [145] showed a 37 bp conserved motif around 50 bp upstream of all pTSSs, except *copG*, *copK*, and *copO* (Figure 7). In repeating this analysis with Improbizer, a 10 bp consensus motif was detected for all detected pTSSs, ca. 60 upstream for most and ca. 70 bp upstream for *copE*, *copL*, *copC*, and *copK*. Palindromic or direct-repeat chromosomal binding sites have been demonstrated for many response regulators of OmpR, NarL, LytR, and PrrA families [146] as well as autoregulation (motif in promoter region of *copR*_1_). Consequently, this could be a putative CopR_1_ binding site. Interestingly, we observed considerable variation in the expression levels of *cop*_1_ genes with similar consensus motifs. This could indicate that these small variations of the operator sequence have large effects on their interaction with regulatory proteins (e.g., CopR_1_), as was shown for binding of response regulator AgrR in *C. metallidurans* NA4 [147], or that additional regulatory factors are involved. In addition, this motif was also detected upstream of the pTSSs of *cusA*, *cupA*, *cupC*, and *copB*_2_ (FIMO; *p* < 0.001 [148]). This discovery implies similarities in the regulation of different Cu-responsive clusters on the chromosome, the chromid, and the pMOL30. Evidently, this putative cross-regulation needs to be validated further.

Several other TSSs were detected in the *cop* cluster. Secondary TSSs were found for *copA*_1_, *copT*, and possibly *copR*_1_. The sTSS at position 191326, likely related to *copA*_1_, was detected in both conditions, but the much stronger pTSS at position 191,243 was only detected in the Cu condition. This observation provides a good example of a Cu-inducible promotor region with a strong signal-to-noise ratio, which can be exploited, e.g., for the construction of optimal Cu-responsive biosensors. Intragenic TSSs were found in 13 of the 21 *cop* genes. Interestingly, the iTSS in *copQ* was expressed more strongly than the pTSS in the CT condition, while the inverse was detected in the Cu condition. This iTSS could potentially lead to an alternative, shortened CDS, where the N-terminal signal peptide as well as one of the typical motifs is absent [82]. Whether this transcript is really translated and has a biological role needs to be further analyzed. Noteworthy, there are 21 *copQ* homologs in *C. metallidurans* CH34, and although this protein family is still poorly characterized, many of them are transcriptionally induced in *C. metallidurans* CH34 in response to different metals [22], and an interplay between different homologues appears to confer metal resistance [82]. Antisense TSSs were identified in *copA*_1_, *copF*, *copH*, *copL*, and *copM*, but only the aTSS in *copL* was overexpressed in the Cu condition, and a motif resembling the 10-bp motif, detected with Improbizer and described above, was detected around the –80 position. The aTSS in *copL* was also validated by 5′/3′ RACE, indicating a transcript size of ± 700 bp (Supplementary Figure S3). The functional role of these putative identified sRNAs in copper resistance will be tested in future studies. In addition to these observations, it is possible that trans-acting sRNAs (see Section 3.2.7) interact with mRNAs from the *cop* cluster.

The third main Cu resistance mechanism on pMOL30 is the HME-RND tripartite efflux pump encoded by *silDCBA*. While none of the *sil* genes are upregulated after acute copper exposure, as discussed previously, we detected several interesting TSSs in this cluster (Figure 6). Two clearly defined TSSs with similar expression levels were found upstream of *silD*, coding for a conserved protein with unknown function. The upstream TSS was preceded by a motif similar to the putative *cop* motif shown in Figure 7, suggesting (partially) shared regulation between these systems. No pTSSs were detected for *silC*, *silB*, or *silA*, indicating that the four-gene *sil* cluster is transcribed as one polycistronic mRNA of over 6 kb in length. A strong iTSS was found at nt 165,562, more or less halfway through the *silA* gene. In addition, we detected a differentially expressed aTSS that could putatively interact with the *silDCBA* mRNA, partially overlapping the ORFs of both *silC* and *silB*. Interestingly, this aTSS was downregulated in the Cu condition, hinting at a role of the antisense transcript in the post-transcriptional regulation of either or both of these two genes. This aTSS was validated by 5′/3′ RACE, indicating a transcript size of ± 500 bp (Supplementary Figure S3). These data could provide insights with regard to the regulation of the *sil* cluster, which has thus far not been elucidated, considering the cluster lacks adjacent proteins with regulatory functions. The possible role of this aTSS will be elucidated in further research.

## 4. Conclusions

In an effort to map the transcriptomic profile and the regulatory features of *C. metallidurans* CH34 in response to acute Cu stress, we performed tagRNA-Seq on *C. metallidurans* CH34 cultures exposed to Cu^2+^ and analyzed both differential gene expression and transcription start sites. Most differentially expressed genes belonged to eggNOG classes representing either transport and metabolism of inorganic ions, transport and metabolism of amino acids, and posttranslational modification, protein turnover, and chaperoning. A considerable fraction of CH34′s impressive HMR arsenal was overexpressed, not limited to Cu-specific detoxification clusters. The importance of the cysteine/sulfur metabolism was also highlighted, in agreement with similar studies. Finally, Cu toxicity via the production of reactive oxygen species (ROS) was detected by the upregulation of multiple clusters involved in cellular redox chemistry. Interestingly, several key differences with previous microarray data were found, likely owing to the lower dose and the longer exposure time used in those experiments. This comparison demonstrates the highly transient nature of the transcriptomic response to Cu stress. Analysis of transcription start sites enabled us to (re)annotate TSSs of over 35% of known CDSs. Many leaderless mRNAs were discovered, often with functions in transcription and replication, recombination, and repair. An unexpectedly high number of TSSs were found inside CDSs, both in sense and antisense. Antisense TSSs were enriched near the 5′ and the 3′ ends of sense transcripts, indicating a function in post-transcriptional regulation of these transcripts. In addition, several transcripts starting in intergenic regions were detected, of which 35 were likely sRNAs. We did not detect sRNAs found in related strains *Burkholderia cenocepacia* [149] and *Bordetella pertussis* [150]. An interesting consensus motif upstream of *cop*_1_ cluster pTSSs was detected, which could be a shared operator of many *cop*_1_ genes. In addition, a substantial number of internal and antisense TSSs were found in this cluster, indicating supplementary levels of regulation. In conclusion, our data not only present new insights in the response of *C. metallidurans* to copper, they also reveal different regulatory aspects that still need to be clarified in order to fully comprehend, optimize, and control possible biotechnological applications of this strain.

## Figures and Tables

**Table 1:** Overview of the main Cu resistance gene clusters in *Cupriavidus metallidurans* CH34.

Gene Cluster	Locus Tag ^1^	Replicon ^2^	Homologous Gene Cluster	Locus Tag ^1^	Replicon ^2^	Function
*copA*_1_ *B*_1_ *C*_1_ *D*_1_	6112–6115	pMOL30	*copA*_2_ *B*_2_ *C*_2_ *D*_2_	5671–5668	CHR2	Periplasmic detoxification
*copF*	6119	pMOL30	*cupA*	3524	CHR1	P_IB1_-type ATPase
*silCBA*	6133–6136	pMOL30	*cusCBA*	5031–5033	CHR2	HME-RND efflux pump

^1^ Locus tag: Rmet_XXXX; ^2^ CHR1: chromosome, CHR2: chromid.

**Table 2:** Number and overlap of detected transcription start sites (TSSs) in each replicon ^1^.

	pTSS			sTSS			iTSS			aTSS			oTSS		
	CT	Cu	∩	CT	Cu	∩	CT	Cu	∩	CT	Cu	∩	CT	Cu	∩
CHR1	1490	1382	1231	729	734	483	1294	1390	916	1015	934	723	278	248	216
CHR2	741	674	586	187	166	102	497	526	359	499	446	348	161	155	134
pMOL28	45	43	38	19	19	13	89	83	74	97	92	77	21	22	18
pMOL30	86	108	78	17	42	11	100	150	76	129	132	101	53	54	46
Genome	2362	2207	1933	952	961	609	1980	2149	1425	1740	1638	1249	513	479	414

^1^ For each replicon and the full genome, the number of TSSs detected in the control (CT) and Cu condition, as well as the intersection (**∩**), are shown per TSS category (primary (pTSS), secondary (sTSS), internal (iTSS), antisense (aTSS) and orphan (oTSS)).

## Supplementary Materials

The following are available online at https://www.mdpi.com/2073-4425/11/9/1049/s1, Table S1: Primers used for 5′ and 3′ RACE experiments, Table S2: MLP output for functional enrichment analysis, Table S3: Data summarization of differential gene expression and transcriptional start site profiling of *C. metallidurans* CH34 in response to Cu^2+^ exposure, Figure S1: General overview of the tagRNA-seq workflow and analysis, Figure S2: Scatter plot of RNA-Seq-derived gene expression of pMOL28 (top) and pMOL30 (bottom) from *C. metallidurans* CH34 exposed for 10 min to 400 µM Cu2+. Dots (blue *p* < 0.05) represent Log2 ratios with red and green lines corresponding to −1 and 1, respectively. CDSs involved in metal resistance are color-coded, Figure S3: Agarose gel images of RACE experiments. Ladder used in all images is GeneRuler 1 kb Plus (Thermo Fischer Scientific). All used primers are described in Supplementary Table S1). Nested PCR as per SMARTER^®^ 5′/3′ RACE User Manual (Takara Bio USA Inc., Mountain View, CA, USA, p. 15–17). Primers used are Universal Primer Short (all reactions) and pasRNA1_5′RACE_NGSP1 (1.1), pasRNA2_5′RACE_NGSP1 (1.2), pasRNA3_5′RACE_NGSP1 (1.3), pasRNA4_5′RACE_NGSP1 (1.4), pasRNA5_5′RACE_NGSP1 (1.5), pasRNA6_5′RACE_NGSP1 (1.6). All amplified sequences were cloned into a pRACE vector and sequenced as described in the 5′/3′ RACE protocol. (2A) Additional nested PCR reactions. Primers used are Universal Primer Short (all reactions) and 3492_o_5′_RACE_NGSP1 (2A.1), 3492_o_3′_RACE_NGSP1 (2A.2), 3492_o_3′_RACE_NGSP2 (2A.3), 3493_o_5′_RACE_NGSP1 (2A.4), 3493_o_3′_RACE_NGSP1 (2A.5), copL_as_5′_RACE_NGSP1 (2A.6), copL_as_3′_RACE_NGSP1 (2A.7), 3616_o_5′_RACE_NGSP1 (2A.8), 3616_o_3′_RACE_NGSP1 (2A.9), pasRNA4_5′RACE_NGSP1 (2A.10), silB_as_3′_RACE_NGSP1 (2A.11). (2B) Control reactions for 5′ and 3′ RACE reactions in (2A), omitting gene-specific primers from the reaction mixture. (2C) Control reactions for 5′ and 3′ RACE reactions in (2A), omitting Universal Primer Short from the reaction mixture, Figure S4: Alignment of original and newly annotated CDSs related to gene clusters involved in metal resistance, Figure S5: SignalP-5.0 output of signal peptide prediction for the re-annotated CopM, Figure S6: Transcription profile analysis of the *cupRAC* cluster from *C. metallidurans* CH34 when exposed to copper. Combined TSS read counts of the three biological replicates for control (upper) and Cu condition (lower) are shown for positive (green) and negative (red) strand, with the *y*-axis containing a break pair (2000–50,000) represented as striped grey lines. CDSs related to the *cupRAC* cluster (coordinates for the chromosome region shown at the bottom) are colored based on their log_2_ fold change. The small black arrows indicate clearly identified primary and internal TSSs. The green arrow represents a re-annotated CDS, Figure S7: Transcription profile analysis of the *czc* cluster from *C. metallidurans* CH34 when exposed to copper. Combined TSS read counts of the three biological replicates for control (upper) and Cu condition (lower) are shown for positive (green) and negative (red) strands, with the *y*-axis containing a break pair (600–2000) represented as striped grey lines. CDSs related to the *czc* cluster (coordinates for the pMOL30 region shown at the bottom) are colored based on their log_2_ fold change. The small black arrows indicate clearly identified primary and internal TSSs. The green arrow represents a re-annotated CDS, Figure S8: Transcription profile analysis of the *pbr* cluster from *C. metallidurans* CH34 when exposed to copper. Combined TSS read counts of the three biological replicates for control (upper) and Cu condition (lower) are shown for positive (green) and negative (red) strands. CDSs related to the *pbr* cluster (coordinates for the pMOL30 region shown at the bottom) are colored based on their log_2_ fold change. The small black arrows indicate clearly identified primary and internal TSSs. The green arrow represents a re-annotated CDS, Figure S9: Consensus motifs (−35 and −10) in *C. metallidurans* CH34 promoters on chromosome (CHR1), chromid (CHR2), and pMOL30, Figure S10: Transcription profile analysis of sigma factors from *C. metallidurans* CH34 when exposed to copper. Combined TSS read counts of the three biological replicates for control (upper) and Cu condition (lower) are shown for positive (green) and negative (red) strands. CDSs are colored based on their log_2_ fold change, Figure S11: Transcription profile analysis of two identified oTSSs (small black arrows; top figure: located on the negative strand of the chromosome at nt 3,784,309; bottom figure: located on the minus strand of the chromid at nt 147,562) from C. metallidurans CH34 when exposed to copper. Combined TSS read counts (red) and combined TSS, PSS, and unassigned read counts (grey, mirrored) of the three biological replicates for control (upper) and Cu condition (lower) are shown for the negative strand. Neighboring CDSs are colored based on their log2 fold change, File TSS_res_finder.py: Python 2 script for the detection of TSSs from coverage files in-depth format with added arguments resolution, replicon length, and coverage cutoff value, File TSS_res_finder_testfile.txt: test file for TSS_res_finder.py with example data.
