# Supplementary material for: The Transcriptomic Landscape of Cupriavidus metallidurans CH34 Acutely Exposed to Copper
Source: Genes (Basel). 2020 Sep 4;11(9):1049. doi: 10.3390/genes11091049 (PMC7563307; doi:10.3390/genes11091049)
Supplement: Supplementary file 1 [file genes-11-01049-s001.zip › Supplementary_materials.docx]

Supplementary Materials

**Supplementary** **Table S1.** Primers used for 5’and 3’ RACE experiments.

| **Primer** | **Sequence** | **TSS position** | **Replicon** | **Genomic context** |
| --- | --- | --- | --- | --- |
| copA_5’RACE_GSP1 | GATTACGCCAAGCTTGCCAAACCTTGTACGAAACGGCG | 191243 | pMOL30 | pTSS of Rmet_6112 |
| copA_5’RACE_NGSP1 | GATTACGCCAAGCTTTGGCAGGTTGGGCAGCAAG | 191243 | pMOL30 | pTSS of Rmet_6112 |
| pasRNA1_5’RACE_GSP1 | GATTACGCCAAGCTTCAGCGACAAAAATGGATGCGGGT | 2045559 | CHR2 | aTSS of Rmet_5332 |
| pasRNA1_5’RACE_NGSP1 | GATTACGCCAAGCTTCTGCTTAACCTTGTGTTGGATATCGAC | 2045559 | CHR2 | aTSS of Rmet_5332 |
| pasRNA2_5’RACE_GSP1 | GATTACGCCAAGCTTCTTCTGAGCAGTCCTGGAAAGCCTACG | 1951037 | CHR2 | aTSS of Rmet_5250 5’UTR |
| pasRNA2_5’RACE_NGSP1 | GATTACGCCAAGCTTGAAAGCCTACGAGCACCCCG | 1951037 | CHR2 | aTSS of Rmet_5250 5’UTR |
| pasRNA3_5’RACE_GSP1 | GATTACGCCAAGCTTACCTTCAACCTGCACCTGAACACCGA | 2102508 | CHR2 | aTSS of Rmet_5375 |
| pasRNA3_5’RACE_NGSP1 | GATTACGCCAAGCTTCCGAGGCGGTGGCGTCA | 2102508 | CHR2 | aTSS of Rmet_5375 |
| pasRNA4_5’RACE_GSP1 | GATTACGCCAAGCTTGGCTGTGGGCCGAGCTCAATTTC | 168587 | pMOL30 | aTSS of Rmet_6135 |
| pasRNA4_5’RACE_NGSP1 | GATTACGCCAAGCTTCAATTTCCTGATGCCGCTTGCGA | 168587 | pMOL30 | aTSS of Rmet_6135 |
| pasRNA5_5’RACE_GSP1 | GATTACGCCAAGCTTGAAGTAAGACTCCCACTCCTCCCAAGC | 73395 | pMOL30 | aTSS of Rmet_6376 |
| pasRNA5_5’RACE_NGSP1 | GATTACGCCAAGCTTGCCACGTGCAGCGCGAA | 73395 | pMOL30 | aTSS of Rmet_6376 |
| pasRNA6_5’RACE_GSP1 | GATTACGCCAAGCTTATGTCGGCTTCGTATGCTTTGGAAATG | 84813 | pMOL30 | aTSS of 3’ end of Rmet_5977 |
| pasRNA6_5’RACE_NGSP1 | GATTACGCCAAGCTTCGGGCGTGCCTGCGTC | 84813 | pMOL30 | aTSS of 3’ end of Rmet_5977 |
| 3492_o_5’_RACE_GSP1 | GATTACGCCAAGCTTCGGGTTCGGTCCAGGCTACTCTTACA | 3784021 | CHR1 | oTSSs 5’ of Rmet_3492 |
| 3492_o_5’_RACE_NGSP1 | GATTACGCCAAGCTTCGCATCGGAGTGTTCTCTCCGCAACT | 3784021 | CHR1 | oTSSs 5’ of Rmet_3492 |
| 3492_o_3’_RACE_GSP1 | GATTACGCCAAGCTTCCCCGCCGCTAAAGCATTGAAAA | 3784021 | CHR1 | oTSSs 5’ of Rmet_3492 |
| 3492_o_3’_RACE_NGSP1 | GATTACGCCAAGCTTGCGCTGAAGAAAGCCCCTCAGGTA | 3784021 | CHR1 | oTSSs 5’ of Rmet_3492 |
| 3492_o_3’_RACE_GSP2 | GATTACGCCAAGCTTGGACCAACCCAGCAGACGTTGCA | 3784104 | CHR1 | oTSSs 5’ of Rmet_3492 |
| 3492_o_3’_RACE_NGSP2 | GATTACGCCAAGCTTCAGGTTGATCCGGAAAACCAGGAAGAC | 3784104 | CHR1 | oTSSs 5’ of Rmet_3492 |
| 3493_o_5’_RACE_GSP1 | GATTACGCCAAGCTTGTAGCCTGGACCGAACCCGTTCCTT | 3784309 | CHR1 | oTSSs 5’ of Rmet_3492 |
| 3493_o_5’_RACE_NGSP1 | GATTACGCCAAGCTTTCGAATTGACCCCTCGAAGGGATTTT | 3784309 | CHR1 | oTSSs 5’ of Rmet_3492 |
| 3493_o_3’_RACE_GSP1 | GATTACGCCAAGCTTGCGCGGACGGGAAAATCCCTT | 3784309 | CHR1 | oTSSs 5’ of Rmet_3492 |
| 3493_o_3’_RACE_NGSP1 | GATTACGCCAAGCTTGGGTCAATTCGAAGGAACGGGTT | 3784309 | CHR1 | oTSSs 5’ of Rmet_3492 |
| copL_as_5’_RACE_GSP1 | GATTACGCCAAGCTTCCCGATATTTACGAGAACGCCGACAA | 181548 | pMOL30 | aTSS of Rmet_6120 |
| copL_as_5’_RACE_NGSP1 | GATTACGCCAAGCTTACCAGCGTGCGTTCGCAAGGTTGCTTCA | 181548 | pMOL30 | aTSS of Rmet_6120 |
| copL_as_3’_RACE_GSP1 | GATTACGCCAAGCTTCTTTGATCCCCACGCCGCTCAGT | 181548 | pMOL30 | aTSS of Rmet_6120 |
| copL_as_3’_RACE_NGSP1 | GATTACGCCAAGCTTGGATCGCGGTGAGCATCTCTGCTT | 181548 | pMOL30 | aTSS of Rmet_6120 |
| 3616_o_5’_RACE_GSP1 | GATTACGCCAAGCTTCTGCCAGGCAACGTTGCCGACCTAGTA | 147562 | CHR2 | oTSS 5’ of Rmet_5941 |
| 3616_o_5’_RACE_NGSP1 | GATTACGCCAAGCTTGGGCAGGGTGATGACGAAGGGGAAGAAT | 147562 | CHR2 | oTSS 5’ of Rmet_5941 |
| 3616_o_3’_RACE_GSP1 | GATTACGCCAAGCTTCACGCCCGAGTCAGCAAATGGGTT | 147562 | CHR2 | oTSS 5’ of Rmet_5941 |
| 3616_o_3’_RACE_NGSP1 | GATTACGCCAAGCTTGACACTTCCCGTGTCCGGCCTTT | 147562 | CHR2 | oTSS 5’ of Rmet_5941 |
| silB_as_3’_RACE_GSP1 | GATTACGCCAAGCTTGCGATTGCGGTCGTTTTCATGGT | 168587 | pMOL30 | aTSS of Rmet_6367 |
| silB_as_3’_RACE_NGSP1 | GATTACGCCAAGCTTGGTTGATCCCCTTGCGTGGATG | 168587 | pMOL30 | aTSS of Rmet_6367 |

**Supplementary Table S2.** MLP output for functional enrichment analysis.

| **eggNOG class** | **Class Description** | **Number of genes in class** | ***p*-value** |
| --- | --- | --- | --- |
| P | Inorganic transport and metabolism | 353 | 7.65*10^-22^ |
| E | Amino acid transport and metabolism | 383 | 0.00276 |
| O | Posttranslational modification, protein turnover, chaperones | 197 | 0.00331 |
| T | Signal transduction mechanisms | 279 | 0.180 |
| K | Transcription | 497 | 0.187 |
| V | Defense mechanisms | 64 | 0.600 |
| G | Carbohydrate transport and metabolism | 201 | 0.739 |
| Q | Secondary metabolites biosynthesis, transport and catabolism | 192 | 0.813 |
| M | Cell wall/membrane/envelope biogenesis | 305 | 0.826 |
| C | Energy production and conversion | 434 | 0.835 |
| H | Coenzyme transport and metabolism | 142 | 0.845 |
| J | Translation, ribosomal structure and biogenesis | 181 | 0.879 |
| F | Nucleotide transport and metabolism | 93 | 0.919 |
| U | Intracellular trafficking, secretion, and vesicular transport | 146 | 0.921 |
| N | Cell motility | 77 | 0.928 |
| I | Lipid transport and metabolism | 287 | 0.934 |
| D | Cell cycle control, cell division, chromosome partitioning | 35 | 0.945 |
| L | Replication, recombination and repair | 334 | 1.000 |


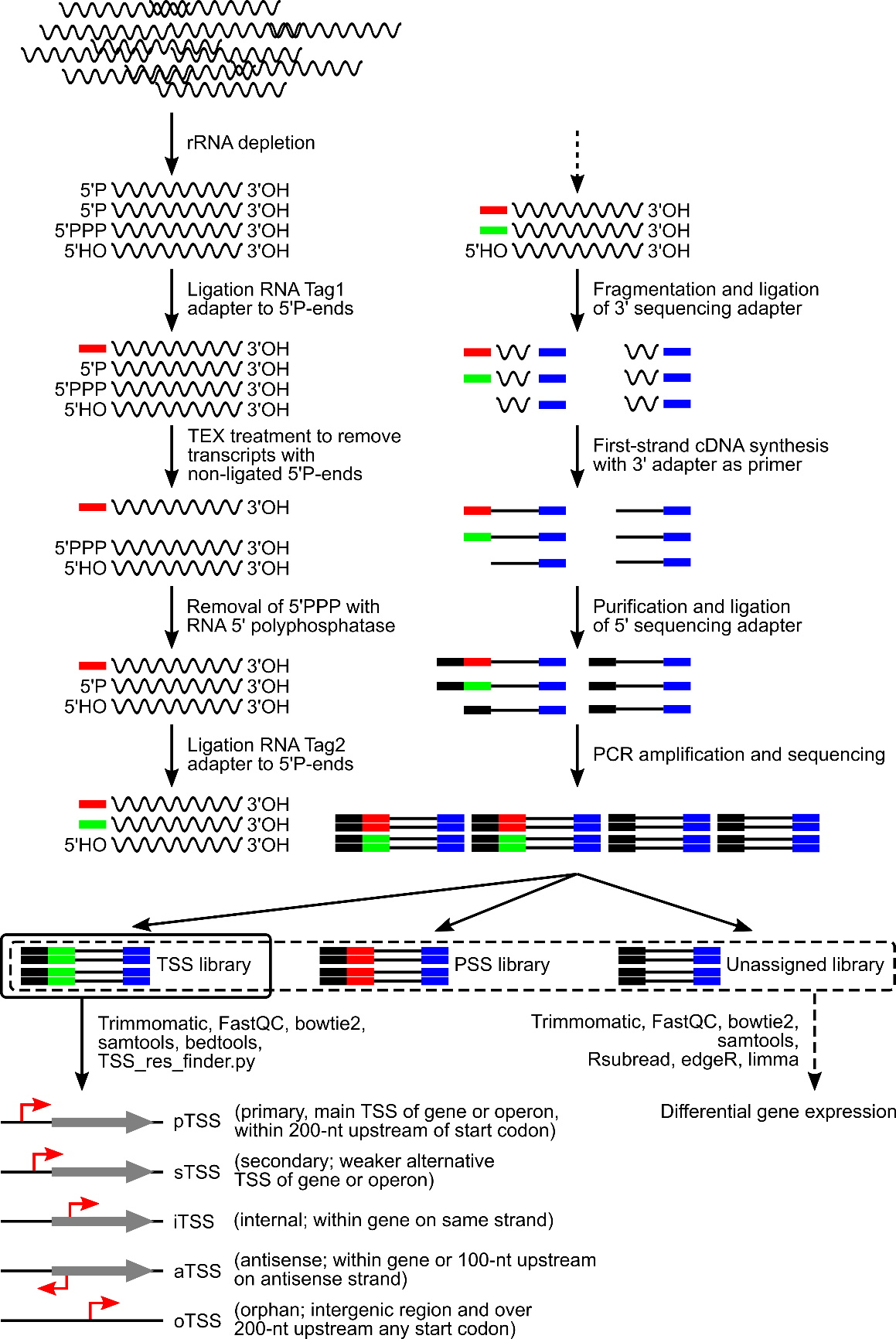


**Supplementary Figure S1.** General overview of the tagRNA-seq workflow and analysis.

*
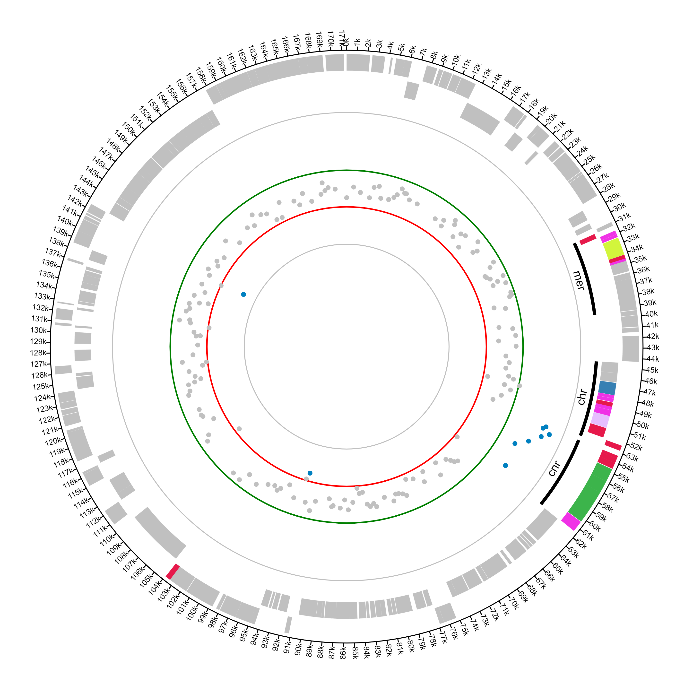
*


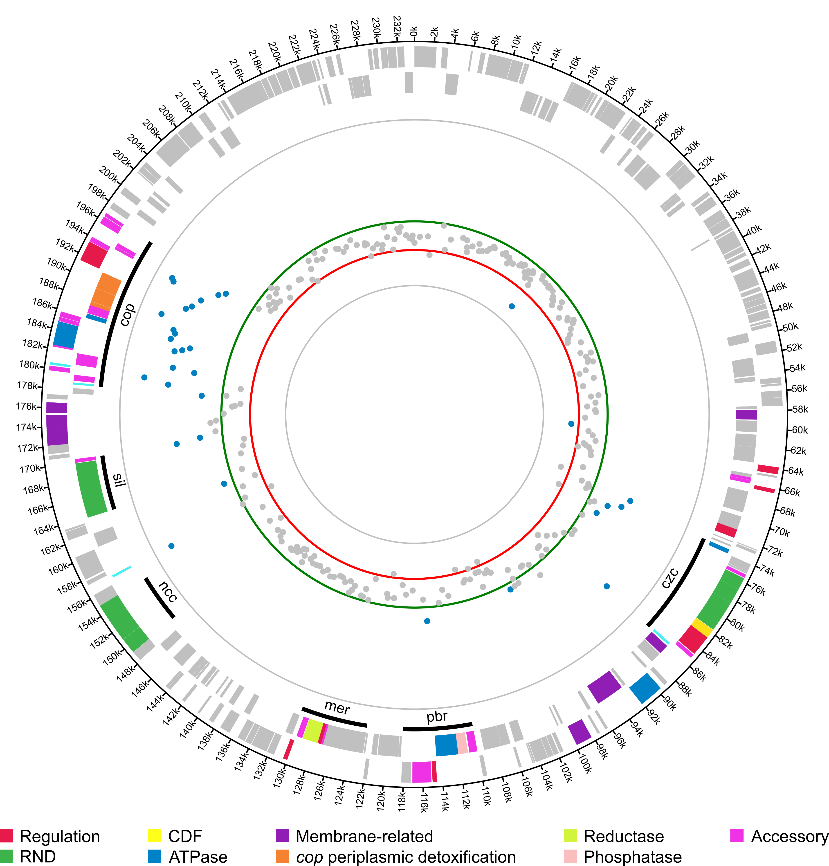


**Supplementary Figure S2.** Scatter plot of RNA-Seq-derived gene expression of pMOL28 (top) and pMOL30 (bottom) from *C. metallidurans* CH34 exposed for 10 min to 400 µM Cu^2+^. Dots (blue p < 0.05) represent Log2 ratios with red and green lines corresponding to -1 and 1, respectively. CDSs involved in metal resistance are color-coded.


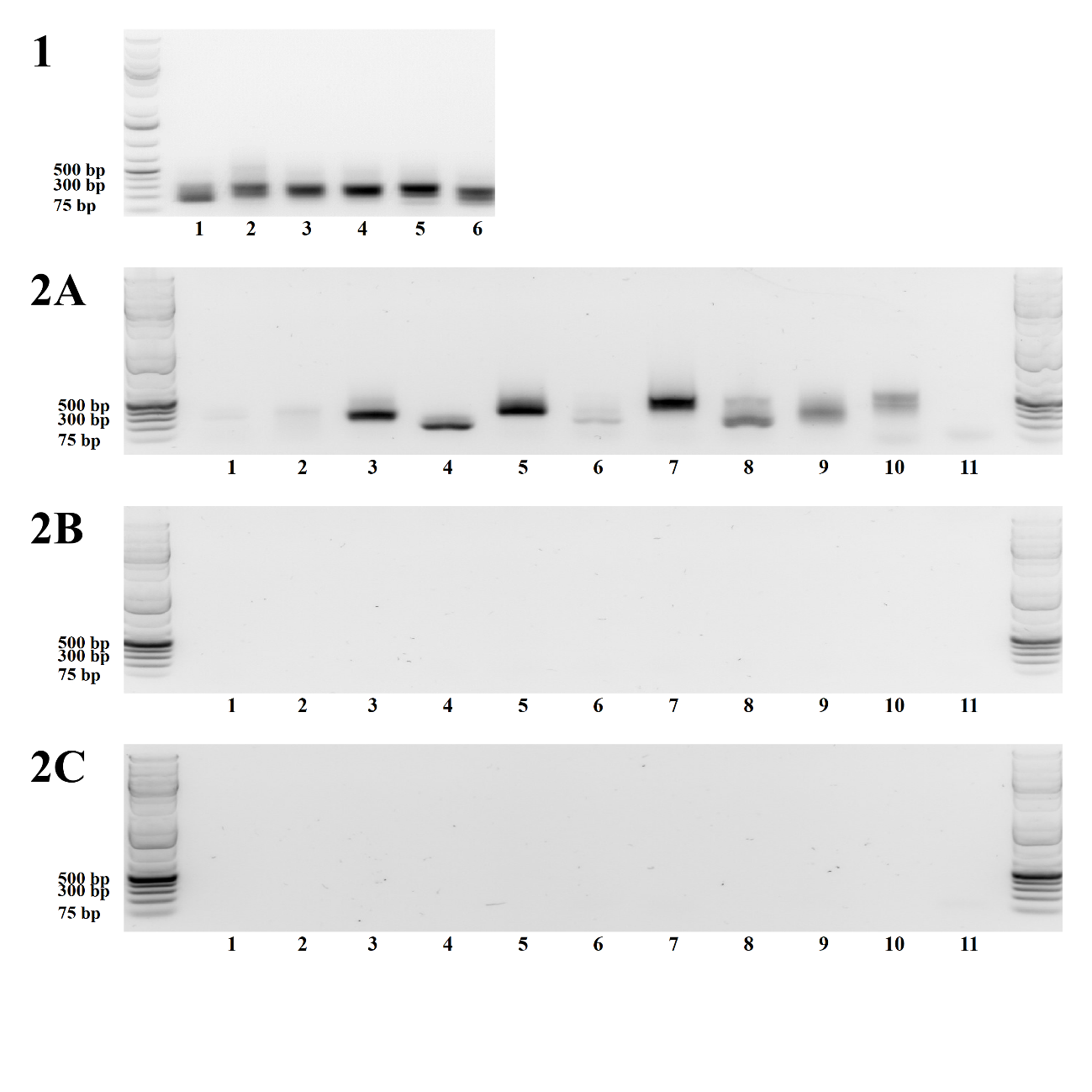


**Supplementary Figure S3.** Agarose gel images of RACE experiments. Ladder used in all images is GeneRuler 1 kb Plus (Thermo Fischer Scientific). All used primers are described in Supplementary Table S1). Nested PCR as per SMARTER® 5’/3’ RACE User Manual (Takara Bio USA Inc., p. 15-17). Primers used are Universal Primer Short (all reactions) and pasRNA1_5’RACE_NGSP1 (1.1), pasRNA2_5’RACE_NGSP1 (1.2), pasRNA3_5’RACE_NGSP1 (1.3), pasRNA4_5’RACE_NGSP1 (1.4), pasRNA5_5’RACE_NGSP1 (1.5), pasRNA6_5’RACE_NGSP1 (1.6). All amplified sequences were cloned into a pRACE vector and sequenced as described in the 5’/3’ RACE protocol. 2A) Additional nested PCR reactions. Primers used are Universal Primer Short (all reactions) and 3492_o_5’_RACE_NGSP1 (2A.1), 3492_o_3’_RACE_NGSP1 (2A.2), 3492_o_3’_RACE_NGSP2 (2A.3), 3493_o_5’_RACE_NGSP1 (2A.4), 3493_o_3’_RACE_NGSP1 (2A.5), copL_as_5’_RACE_NGSP1 (2A.6), copL_as_3’_RACE_NGSP1 (2A.7), 3616_o_5’_RACE_NGSP1 (2A.8), 3616_o_3’_RACE_NGSP1 (2A.9), pasRNA4_5’RACE_NGSP1 (2A.10), silB_as_3’_RACE_NGSP1 (2A.11). 2B) Control reactions for 5’ and 3’ RACE reactions in (2A), omitting gene-specific primers from the reaction mixture. 2C) Control reactions for 5’ and 3’ RACE reactions in (2A), omitting Universal Primer Short from the reaction mixture.


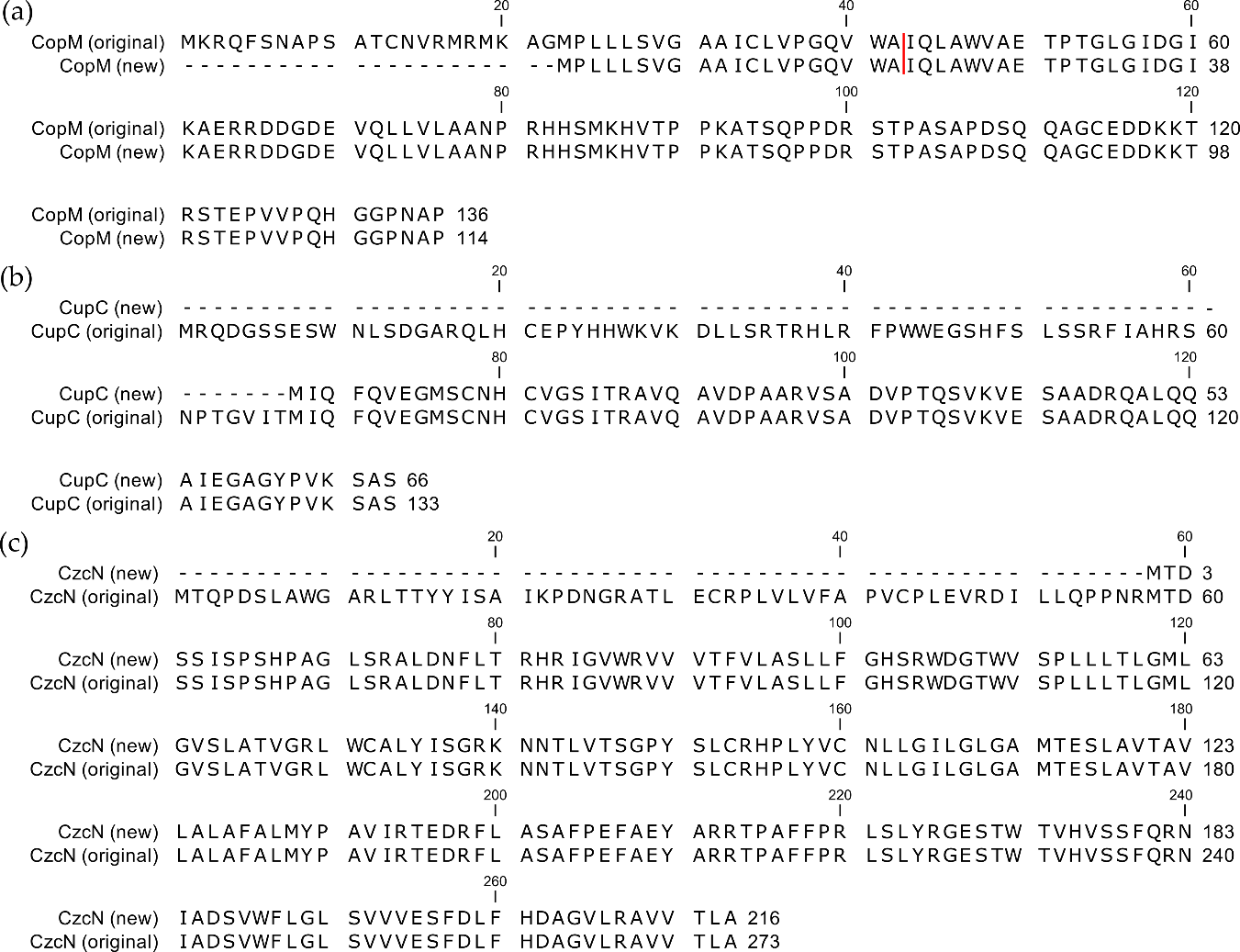


**Supplementary Figure S4.** Alignment of original and newly annotated CDSs related to gene clusters involved in metal resistance.


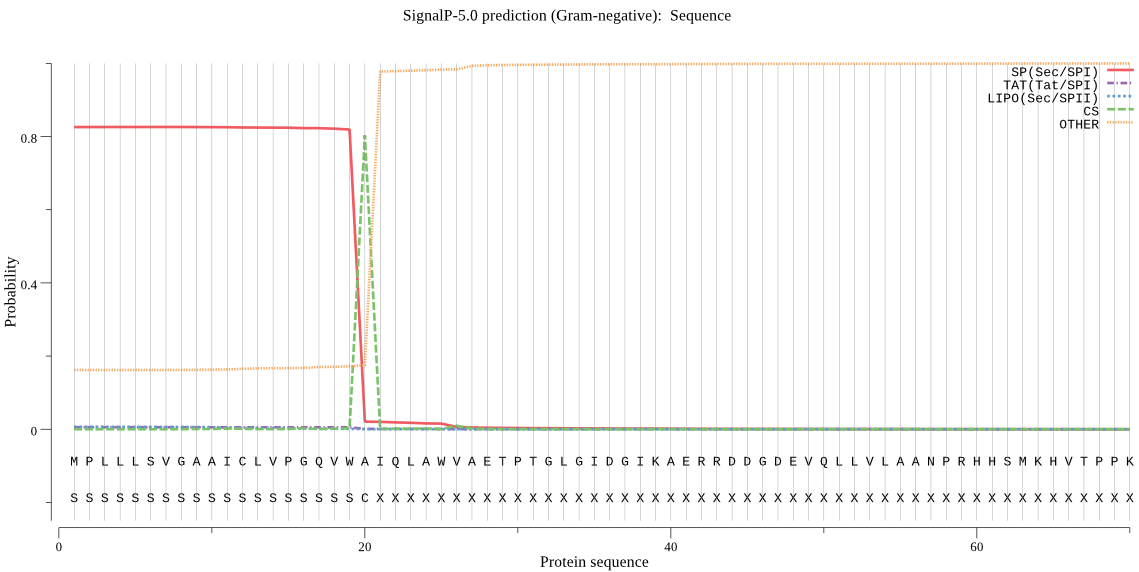


**Supplementary Figure S5.** SignalP-5.0 output of signal peptide prediction for the reannotated CopM.


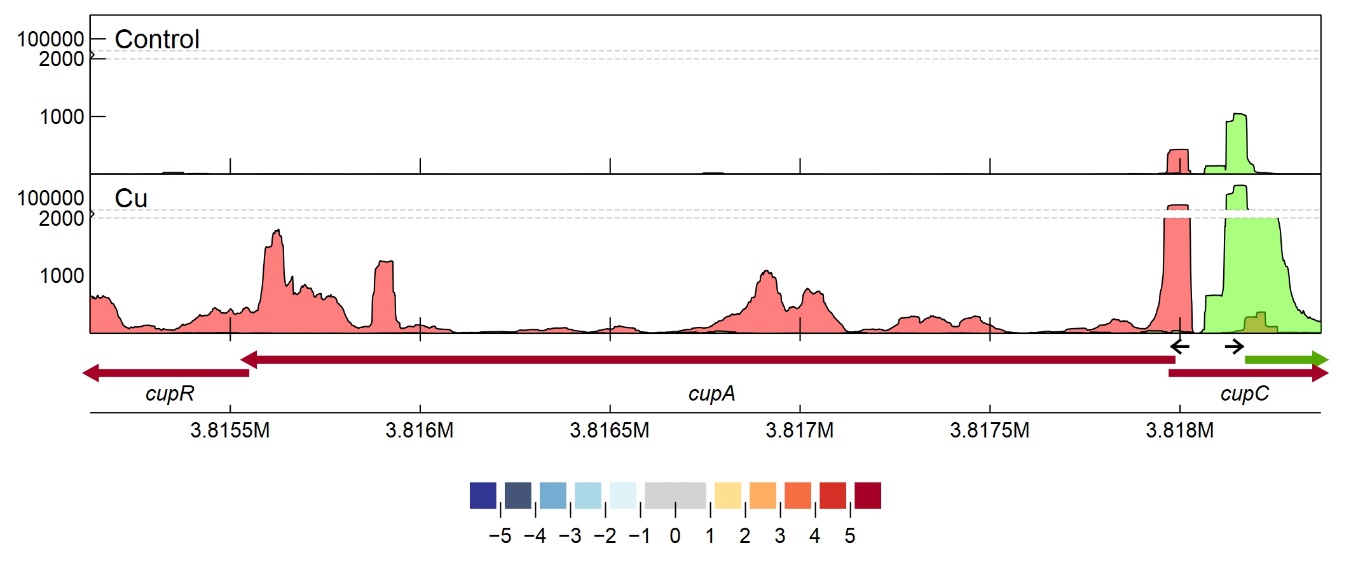


**Supplementary Figure S6.** Transcription profile analysis of the *cupRAC* cluster from *C. metallidurans* CH34 when exposed to copper. Combined TSS read counts of the three biological replicates for control (upper) and Cu condition (lower) are shown for the positive (green) and negative (red) strand, with the y-axis containing a break pair (2000-50000) represented as striped grey lines. CDSs related to the *cupRAC* cluster (coordinates for the chromosome region shown at the bottom) are colored based on their log_2_ fold change. The small black arrows indicate clearly identified primary and internal TSSs. The green arrow represents a re-annotated CDS.


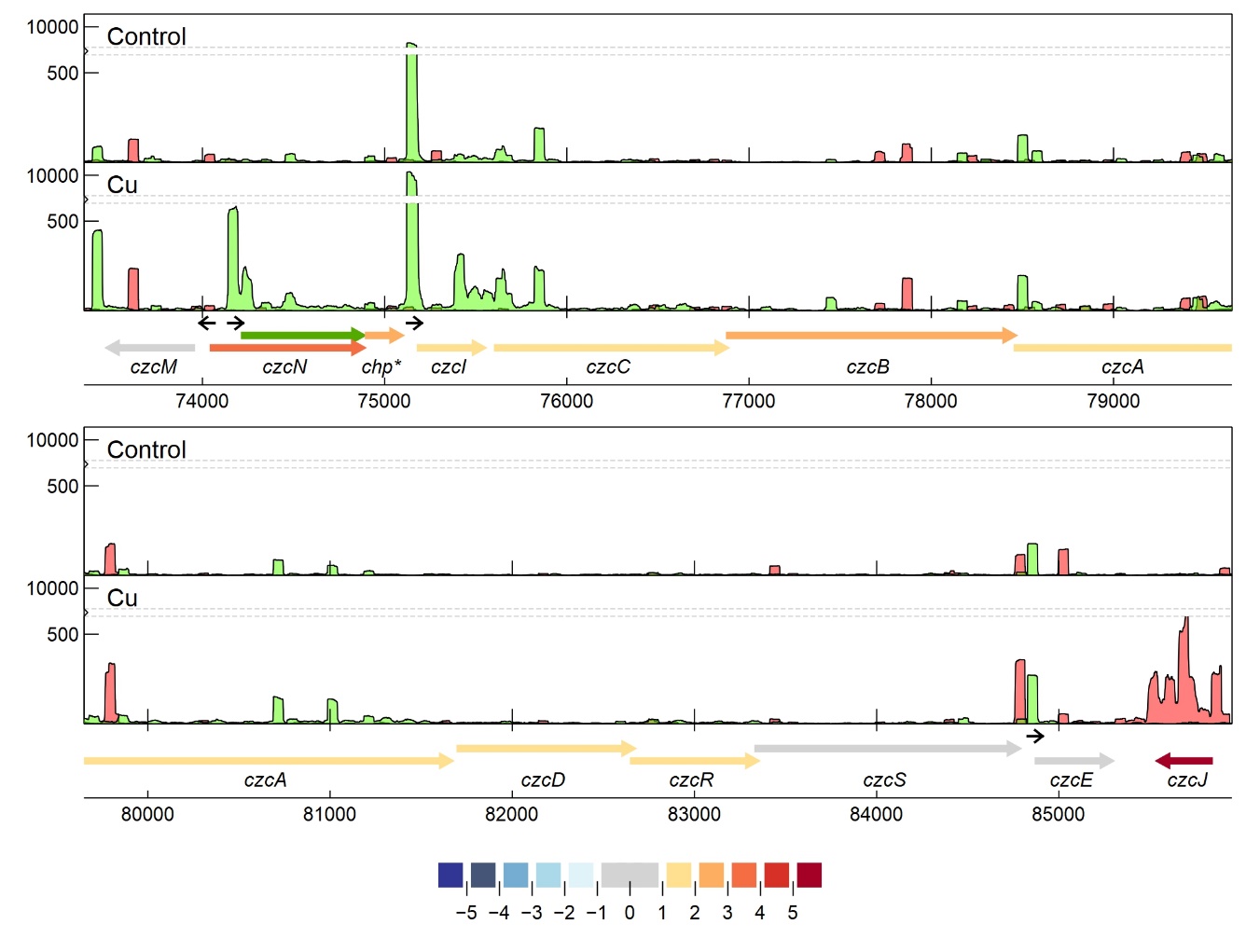


**Supplementary Figure S7.** Transcription profile analysis of the *czc* cluster from *C. metallidurans* CH34 when exposed to copper. Combined TSS read counts of the three biological replicates for control (upper) and Cu condition (lower) are shown for the positive (green) and negative (red) strand, with the y-axis containing a break pair (600-2000) represented as striped grey lines. CDSs related to the *czc* cluster (coordinates for the pMOL30 region shown at the bottom) are colored based on their log_2_ fold change. The small black arrows indicate clearly identified primary and internal TSSs. The green arrow represents a re-annotated CDS.


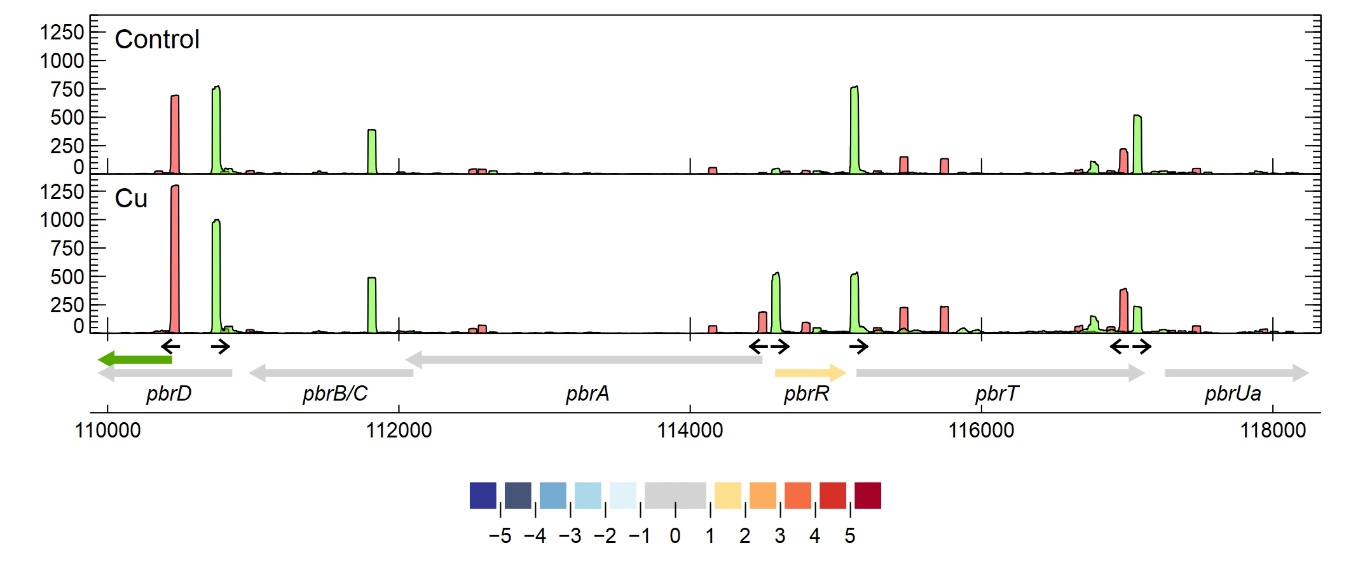


**Supplementary Figure S8.** Transcription profile analysis of the *pbr* cluster from *C. metallidurans* CH34 when exposed to copper. Combined TSS read counts of the three biological replicates for control (upper) and Cu condition (lower) are shown for the positive (green) and negative (red) strand. CDSs related to the *pbr* cluster (coordinates for the pMOL30 region shown at the bottom) are colored based on their log_2_ fold change. The small black arrows indicate clearly identified primary and internal TSSs. The green arrow represents a re-annotated CDS.

| 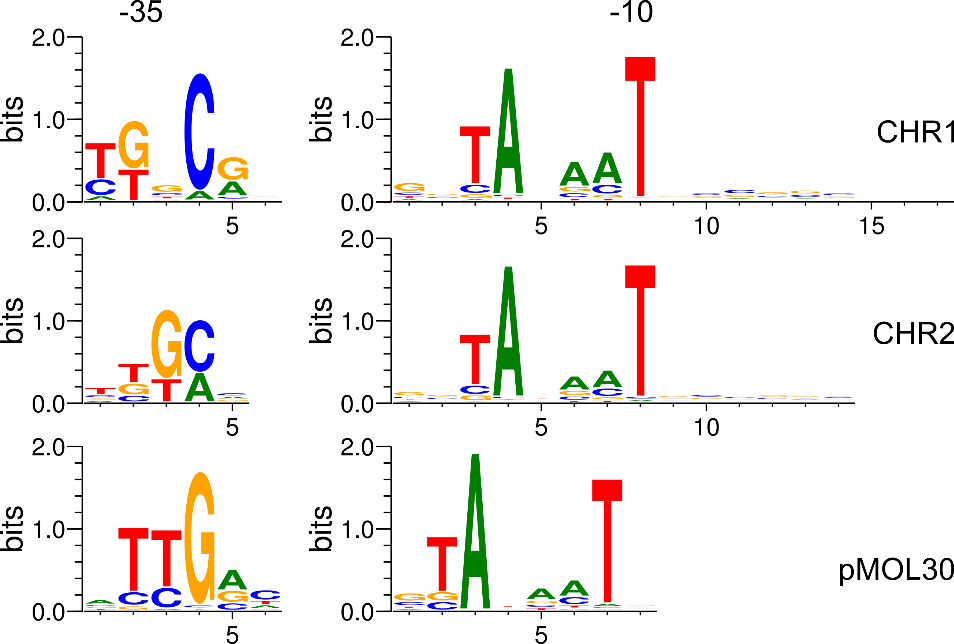 |
| --- |

**Supplementary Figure S9.** Consensus motifs (-35 and -10) in *C. metallidurans* CH34 promoters on the chromosome (CHR1), chromid (CHR2) and pMOL30.


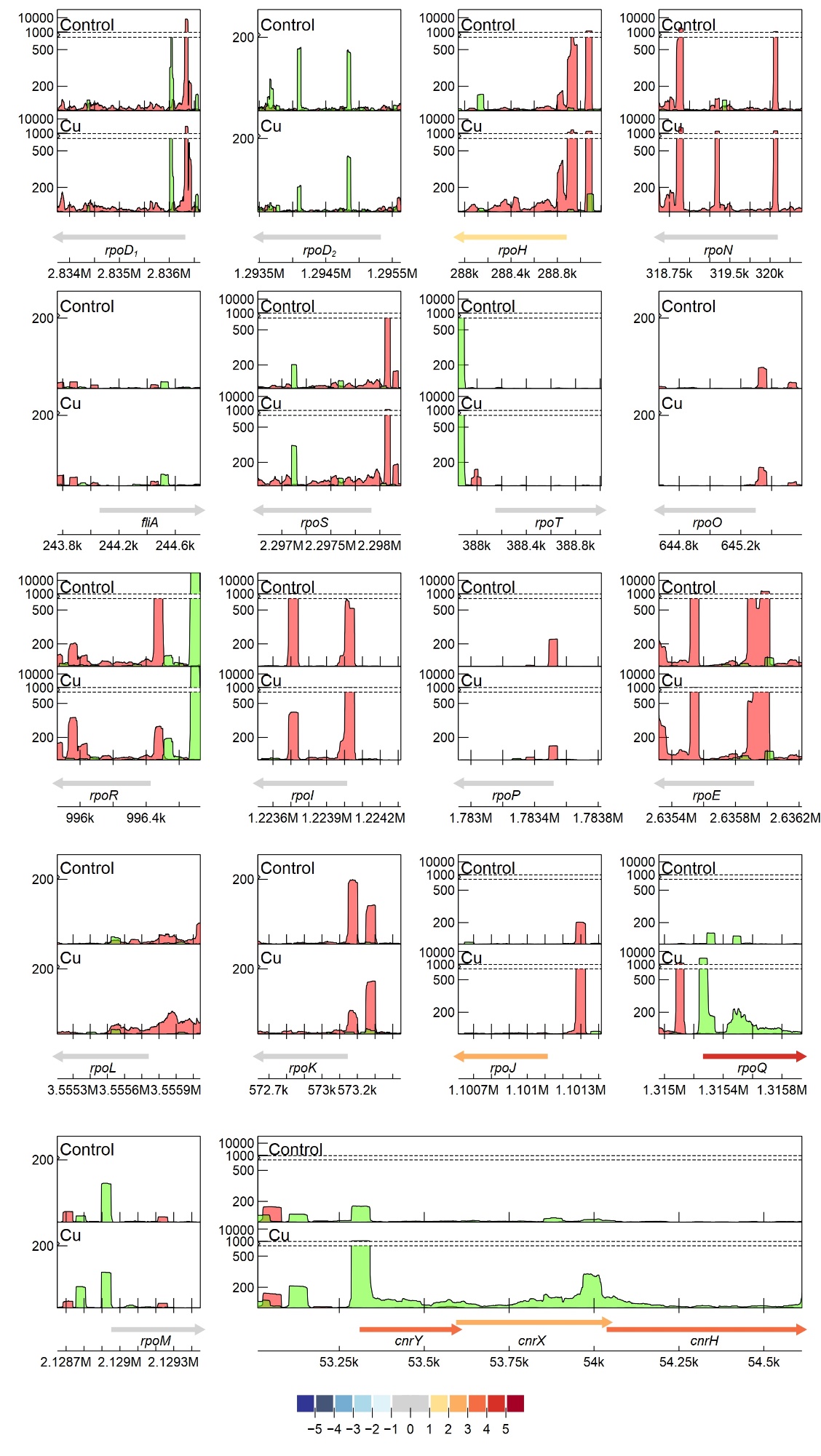


**Supplementary Figure S10.** Transcription profile analysis of sigma factors from *C. metallidurans* CH34 when exposed to copper. Combined TSS read counts of the three biological replicates for control (upper) and Cu condition (lower) are shown for the positive (green) and negative (red) strand. CDSs are colored based on their log_2_ fold change.

**
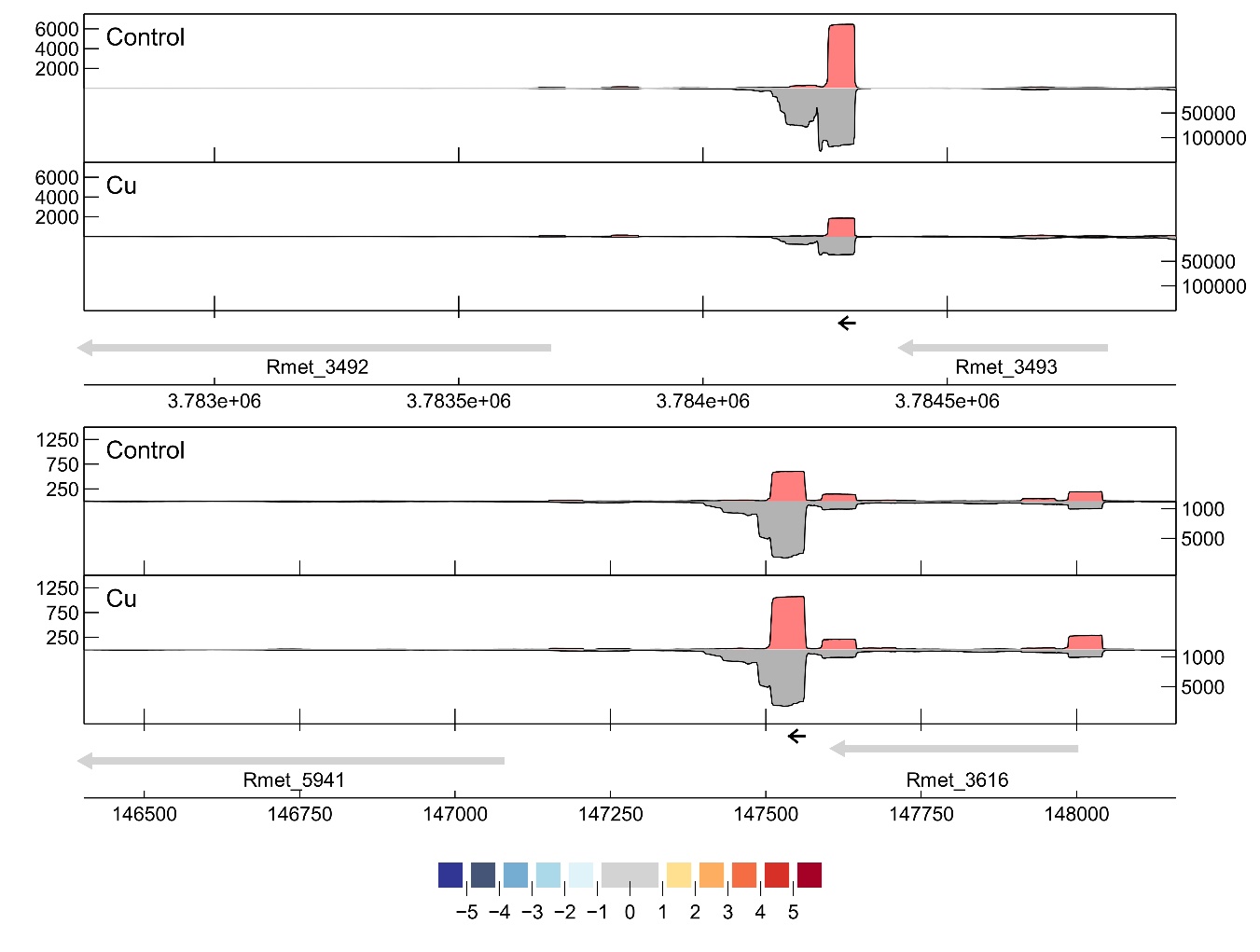
**

**Supplementary Figure S11.** Transcription profile analysis of 2 identified oTSSs (small black arrows; top figure: located on the negative strand of CHR1 at nt 3784309; bottom figure: located on the minus strand of CHR2 at nt 147562) from *C. metallidurans* CH34 when exposed to copper. Combined TSS read counts (red) and combined TSS, PSS and unassigned read counts (grey, mirrored) of the three biological replicates for control (upper) and Cu condition (lower) are shown for the negative strand. Neighboring CDSs are colored based on their log_2_ fold change.
